# Multimodal Evaluation of the Anti-Adhesion Effects of Heparin-Loaded PLGA/HA Nanofiber Membranes in an Intraperitoneal Adhesion Model in Rats

**DOI:** 10.3390/ijms27198715

**Published:** 2026-09-29

**Authors:** Turgay Karatas, Nurcan Gokturk, Kubra Karadas Gedik, Burak Karabulut, Damla Bilge Tasdemir, Mehmet Akif Turkoz, Ahmet Ulu, Yusuf Turkoz, Necati Timurkaan, Tugba Dogan, Anıl Kaya, Engin Burak Selcuk, Mehmet Karatas, Meryem Budak, Burhan Ates

**Affiliations:** 1 Department of Anatomy, Faculty of Medicine, Inonu University, Malatya 44050, Türkiye; 2 Department of Medical Biochemistry, Faculty of Medicine, Inonu University, Malatya 44050, Türkiye; 3 Department of Chemistry, Faculty of Arts and Sciences, Inonu University, Malatya 44050, Türkiye; 4 Department of Pathology, Faculty of Veterinary Medicine, Fırat University, Elazığ 23119, Türkiye; 5 Department of Radiology, Faculty of Medicine, Inonu University, Malatya 44050, Türkiye; 6 Department of Anatomy, Faculty of Medicine, Malatya Turgut Özal University, Malatya 44210, Türkiye; 7 Department of Family Medicine, Faculty of Medicine, Inonu University, Malatya 44050, Türkiye; 8 Department of Medical History and Ethics, Faculty of Medicine, Inonu University, Malatya 44050, Türkiye; 9 Faculty of Dentistry, Bingöl University, Bingöl 12000, Türkiye

**Keywords:** intraabdominal adhesion, electrospinning, PLGA/HA nanofiber membrane, heparin, drug delivery

## Abstract

This study aimed to produce and characterize nanofiber membranes (PLGA/HA@LMWH) containing Poly(lactic-co-glycolic acid)/Hyaluronic Acid (PLGA/HA) immobilized with low-molecular-weight heparin (LMWH), and to investigate their physical and chemical effects in preventing intraabdominal adhesions in a rat model using multidisciplinary methods. Forty female Wistar Albino rats were divided into four groups (*n* = 10). Cecal abrasions were induced in three groups: treated with PLGA/HA membrane, PLGA/HA@LMWH membrane, and 0.9% NaCl (SS). The fourth group underwent laparotomy only. On day 21, rats were sacrificed and evaluated macroscopically, histopathologically, immunohistochemically, and biochemically. Macroscopic adhesion, inflammation, fibrosis, neovascularization, collagen scores, and TGF-β1 expression were found to be high in the SS and PLGA/HA groups, while a significant decrease (*p* < 0.05–0.01) was detected in all of these values in the PLGA/HA@LMWH group. NF-κB activation was significantly decreased in the PLGA/HA and especially the PLGA/HA@LMWH groups compared to the SS group (*p* < 0.05). In addition, apoptosis was suppressed in the PLGA/HA@LMWH group, with increased Bcl-2 and decreased cleaved caspase-3. The PLGA/HA@LMWH membrane reduced peritoneal adhesions by suppressing inflammation, fibrosis, neovascularization, and collagen deposition, lowering TGF-β1 expression and NF-κB and p-NF-κB levels, and suppressing apoptosis. However, large-scale studies are needed for clinical use.

## 1. Introduction

Intra-abdominal adhesions are pathological attachments of organ surfaces or peritoneal layers to each other following abdominal or pelvic surgery, inflammatory processes, radiation, or trauma [1,2,3]. Despite the development of various barrier biomaterials and active pharmacological agents, peritoneal adhesions continue to represent a significant clinical challenge. Indeed, even with modern surgical techniques, adhesion formation is reported in over 90% of patients undergoing major abdominal surgery [4,5,6,7].

Although peritoneal adhesions are generally asymptomatic, they can lead to serious problems such as small bowel obstruction, chronic pain, infertility, and surgical difficulties [3,4,7,8,9,10]. While increasing operation time and intraoperative risks, mortality in bowel obstruction reaches 7.2% [8]. In the US, the annual economic cost exceeds $ 1.3 billion [4], and in addition to the billions of dollars it places on healthcare systems, it also causes significant legal liabilities [11,12].

Mesothelial cells in the peritoneum provide lubrication between surfaces [4]. Under physiological conditions, these cells prevent fibrin accumulation. In cases such as surgical trauma, ischemia, or infection, peritoneal injury triggers inflammation and coagulation, leading to the formation of fibrin-rich exudate. Peritoneal mesothelial cells are thought to play a significant role in regulating peritoneal fibrinolytic activity by producing the majority of tissue plasminogen activator (tPA) and plasminogen activator inhibitor-1 (PAI-1) [7,8,13].

Adhesion formation begins with inflammation following surgical trauma, increased vascular permeability, and activation of the coagulation cascade [14]. The resulting fibrous exudate establishes a fibrin network between the damaged surfaces [15,16,17]. Increased PAI-1 and decreased t-PA activity inhibit fibrin degradation, leading to the persistence of the fibrin matrix; this matrix transforms into fibrotic adhesions through fibroblast proliferation and collagen deposition [8,10,11,15].

In current practice, adhesion prevention relies on surgical technique optimization and the use of barrier agents [18]. Minimally invasive surgery, careful hemostasis, reduction in tissue trauma and prevention of ischemia are not sufficient on their own; therefore, the use of barrier agents is gaining importance [9,18,19]. The first 5–7 days are critical for adhesion formation, and the anti-adhesive barriers prevent fibrinous adhesions by separating damaged surfaces during this period. The most common methods are physical barriers such as membranes, gels, and liquids [20,21,22]. However, the success rates of commonly used membranes (Seprafilm^®^ Adhesion Barrier (Baxter Healthcare Corporation, Deerfield, IL, USA), Interceed^®^ Absorbable Adhesion Barrier (Ethicon, Inc. Neuchâtel, Switzerland) are low due to the difficulty and fragility of their application in laparoscopy, and liquid barriers are also low due to their short duration of action [1,19,20,22,23].

The effectiveness of existing barriers is limited, and innovative strategies combining structural and pharmacological properties are needed to prevent adhesion. Electrospinning nanofiber membranes offer local solutions depending on the polymer type; with their large surface area and porous structure, they are widely used as physical barriers to prevent adhesion [24,25]. The use of these membranes as barriers requires them to be biocompatible, have high mechanical strength, be easy to apply, and have a wettable surface. Furthermore, electrospinning-produced structures must be biodegradable and support tissue regeneration. Synthetic biopolymers [e.g., Poly-lactic-co-glycolic acid (PLGA)] are frequently preferred in tissue engineering due to their biocompatibility, tunable biodegradability, and mechanical performance. Natural glycosaminoglycan hyaluronic acid (HA), on the other hand, supports tissue regeneration and modulates inflammation [21,26].

Current adhesion prevention strategies focus on the functionalization of barriers with pharmacological agents. In this context, Low-Molecular-Weight Heparin (LMWH) stands out with its anticoagulant, anti-inflammatory, anti-fibrotic, and angiogenesis-modulating effects. LMWH prevents adhesion formation by inhibiting coagulation and suppressing fibrin deposition and inflammation [27]. It also modulates the activity of profibrotic mediators such as TGF-β. This multifaceted biological activity makes heparin an effective candidate for the functionalization of adhesion prevention barriers [28]. LMWH inhibits thrombin formation with antithrombin III and supports the fibrinolytic process [12]. Although free heparin carries a risk of bleeding, local and controlled release from nanofiber membranes minimizes these side effects [29].

Our hypothesis is that LMWH-loaded PLGA/HA nanofiber membranes significantly reduce adhesion formation in a rat intraperitoneal adhesion model, and that this effect will arise from the synergistic interaction between the membrane’s physical barrier function and the anticoagulant and anti-inflammatory properties of heparin. Furthermore, it is anticipated that the findings obtained from this study will elucidate the potential of nanotechnology-based, pharmacologically functionalized biomaterials in the prevention of intra-abdominal adhesions and provide a scientific basis for future clinical investigations.

The aim of this study is to characterize PLGA/HA nanofiber membranes produced by electrospinning, to functionalize these membranes with LMWH to create a PLGA/HA@LMWH formulation, and to investigate the intraabdominal adhesion-preventing efficacy of the membranes in a rat model.

## 2. Results

### 2.1. Produced Antiadhesive Materials

#### 2.1.1. Characterization Results

**Figure 1:**
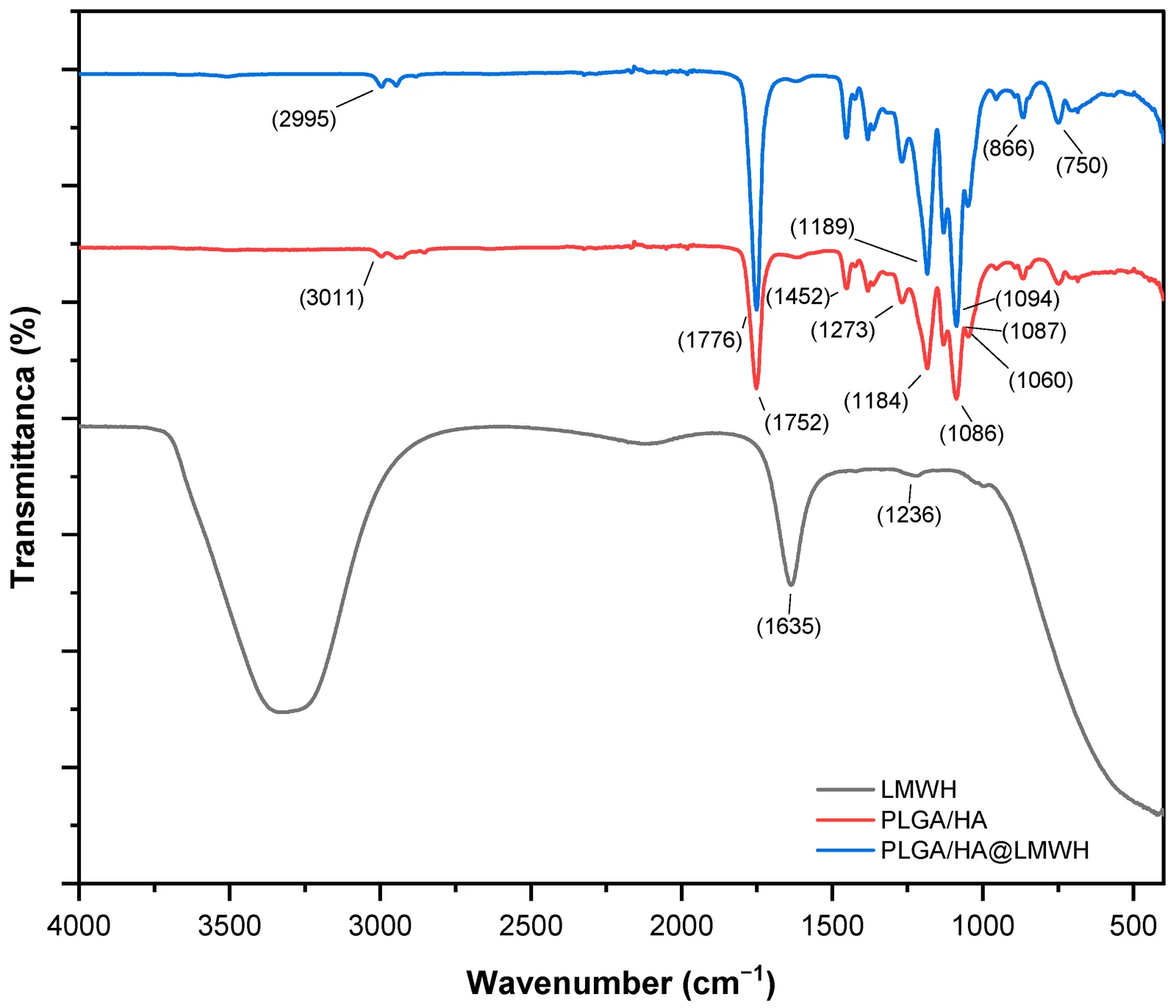
FT-IR spectra of LMWH, PLGA/HA, and PLGA/HA@LMWH nanofibers.

Fourier-transform infrared spectroscopy (FT-IR) spectra showing the intermolecular interactions of PLGA/HA and PLGA/HA@LMWH nanofibers are presented in Figure 1. The spectra of pure LMWH, PLGA/HA, and PLGA/HA@LMWH nanofiber membranes were evaluated comparatively. In the pure LMWH spectrum, the C–O–S vibrations with asymmetric S=O at 1220–1260 cm^−1^ and symmetric S=O at 1030–1080 cm^−1^ indicate the sulfated structure of heparin. In the PLGA/HA nanofiber spectrum, PLGA’s ~1755 cm^−1^ ester C=O and HA’s 1600–1650 cm^−1^ amide bands were observed, while sulfate bands were absent. Upon addition of LMWH, the characteristic PLGA and HA bands were preserved in the PLGA/HA@LMWH spectrum, and LMWH bands of 1220–1260 and 1030–1080 cm^−1^ became prominent. The sulfate ester bands in the range of 840–850 cm^−1^ demonstrate that LMWH was incorporated into the nanofiber matrix while preserving its chemical structure [30,31,32].

**Figure 2:**
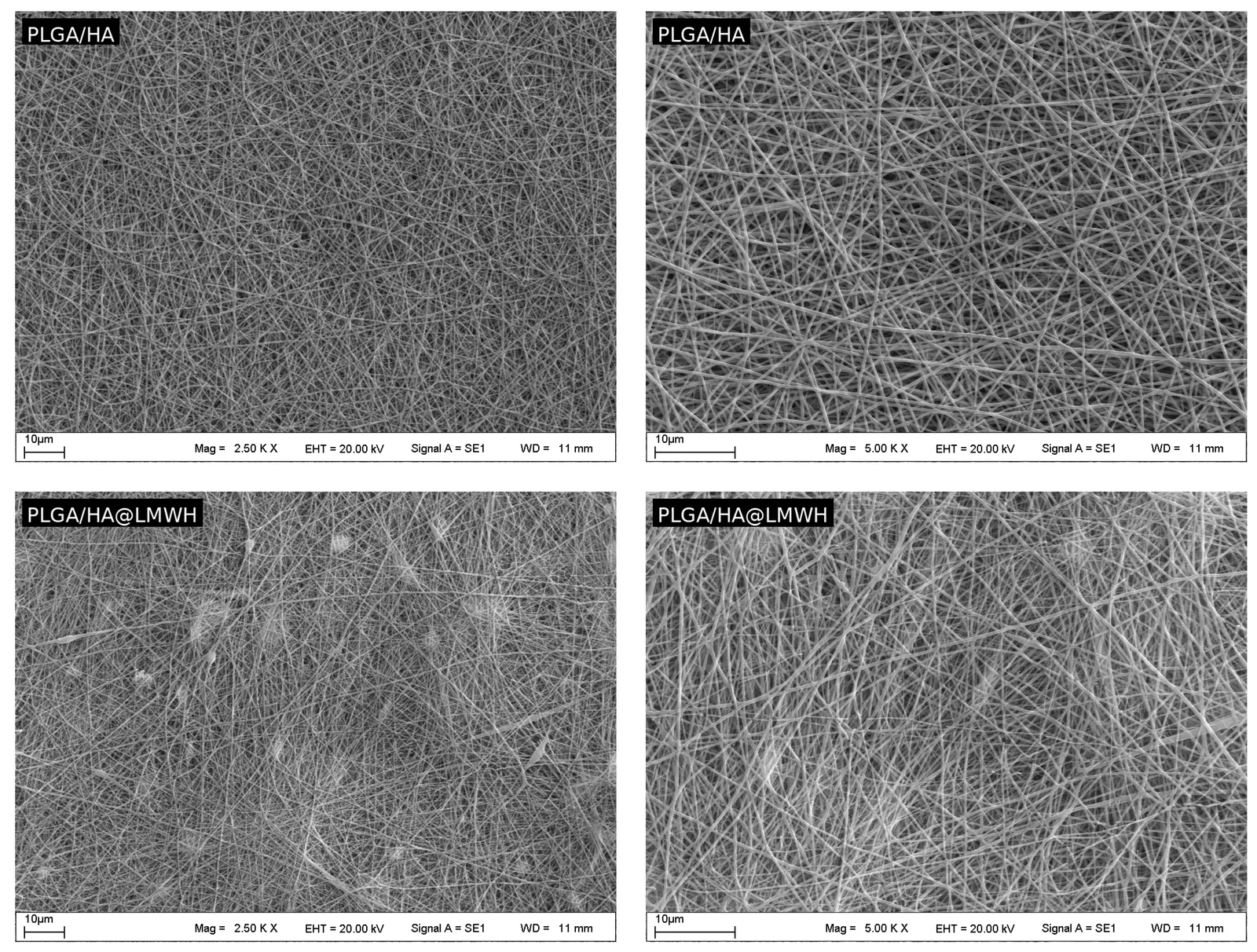
SEM images of PLGA/HA and PLGA/HA@LMWH nanofibers at 2.50 KX and 5.00 KX magnifications.

To evaluate the overall fibrous mesh morphology and surface characteristics, lower magnification SEM micrographs (2500× and 5000×) were acquired for both PLGA/HA and PLGA/HA@LMWH membranes (Figure 2). The microstructural analysis indicated that both groups formed an interconnected, continuous non-woven nanofibrous network. While SEM imaging shows that LMWH incorporation maintained the overall structural integrity and nanofiber network without macroscopic phase separation, the elemental and molecular integration of heparin into the nanofibers was further analyzed through energy-dispersive X-ray spectroscopy (EDX) (presence of sulfur, S) and X-ray diffraction (XRD) analyses.

**Figure 3:**
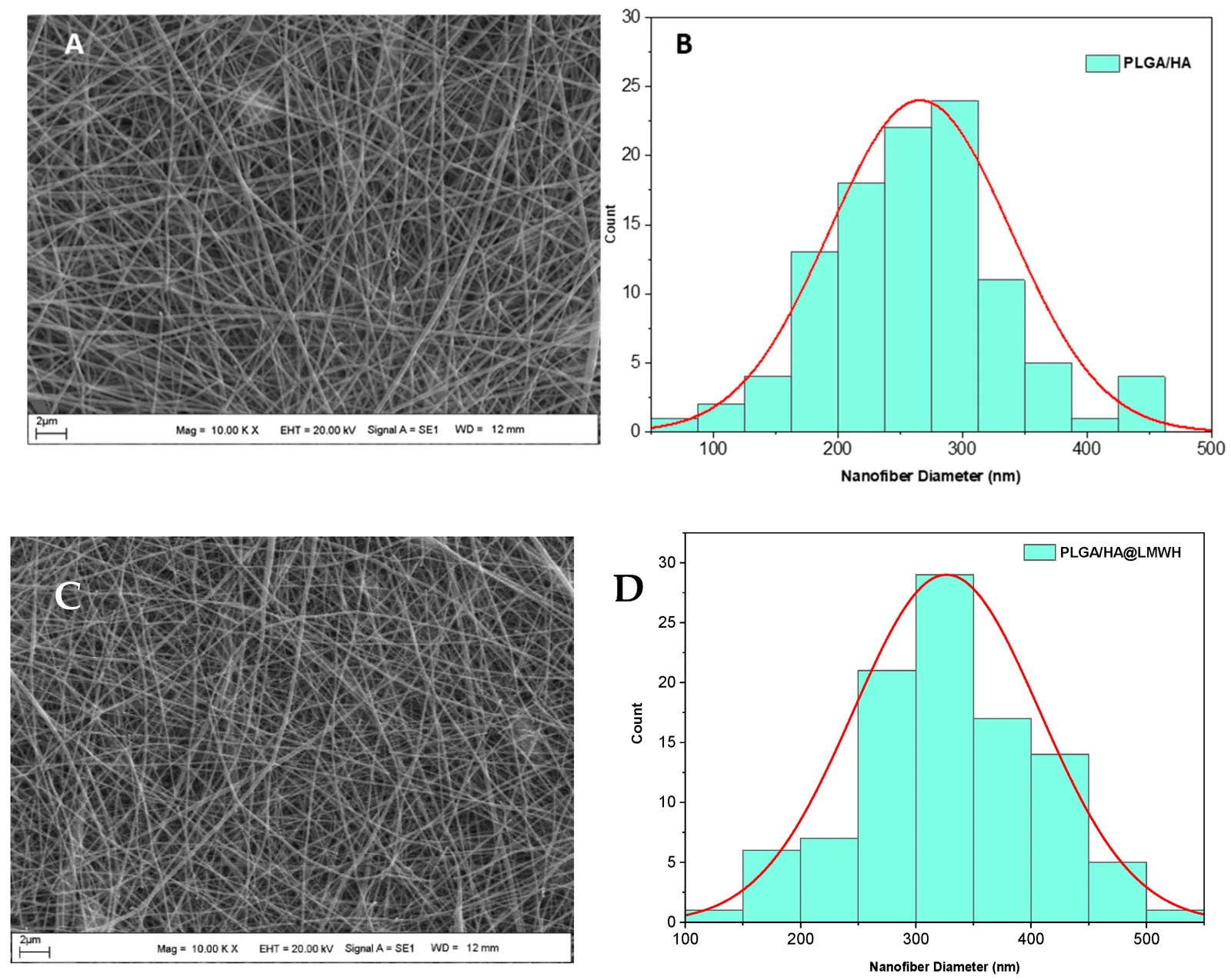
SEM images and fiber diameter distribution histograms of PLGA/HA (**A**,**B**) and PLGA/HA@LMWH nanofibers (**C**,**D**). The red curve represents the fitted distribution curve of the fiber diameters.

Following the overall morphological assessment, Figure 3 presents higher-magnification scanning electron microscopy (SEM) images and fiber diameter distributions of PLGA/HA and PLGA/HA@LMWH membranes. The randomly oriented nanofiber network observed in Figure 3A indicates the formation of continuous electrospun fibers. The fiber diameter histogram in Figure 3B shows that PLGA/HA nanofibers exhibit a distribution with an average diameter of 290 ± 5 nm. This fiber distribution is consistent with the literature reports on the processing of PLGA-based systems [33,34,35].

The SEM image in Figure 3C indicates that the PLGA/HA@LMWH nanofibers preserve their overall fibrous network structure upon heparin addition. According to Figure 3D, the average fiber diameter was determined to be 320 ± 5 nm with the addition of heparin. Consequently, both membranes demonstrated morphological features suitable for barrier applications, such as a high surface area-to-volume ratio and an interconnected porous structure.

**Figure 4:**
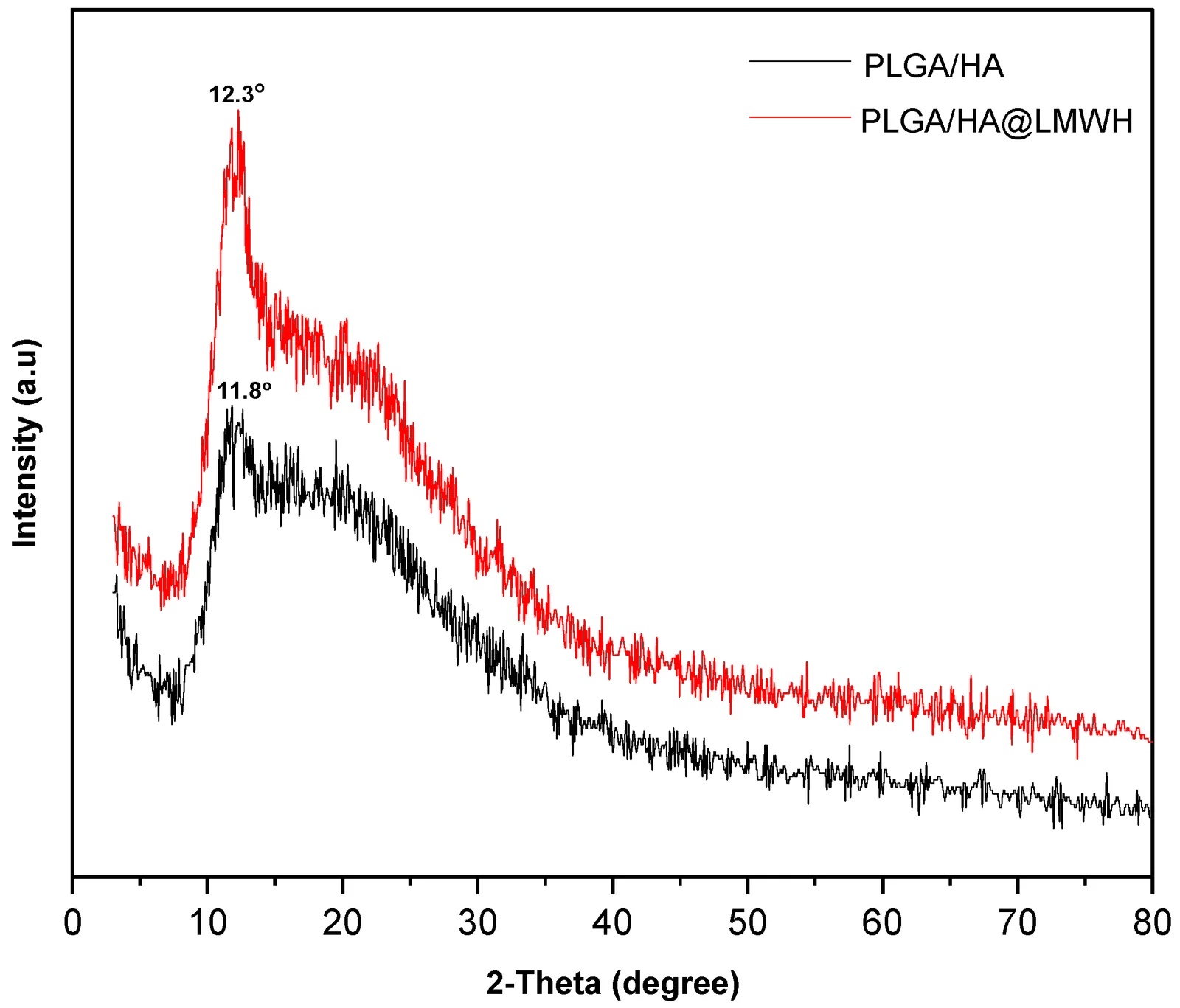
XRD spectra of PLGA/HA and PLGA/HA@LMWH nanofibers.

The crystalline/amorphous state of the electrospun membranes was investigated by XRD analysis to evaluate the structural incorporation of LMWH. As presented in Figure 4, both the PLGA/HA and PLGA/HA@LMWH nanofiber scaffolds exhibited a broad amorphous halo in the range of 2 theta = 10–25°. A characteristic broad peak was identified around 2 theta = 11.8° for the PLGA/HA nanofibers. With the incorporation of low-molecular-weight heparin (PLGA/HA@LMWH), this broad diffraction maximum shifted to 12.3° with a small change in intensity. This confirms that LMWH is molecularly dispersed in an amorphous state rather than forming crystalline aggregates within the PLGA/HA matrix.

**Figure 5:**
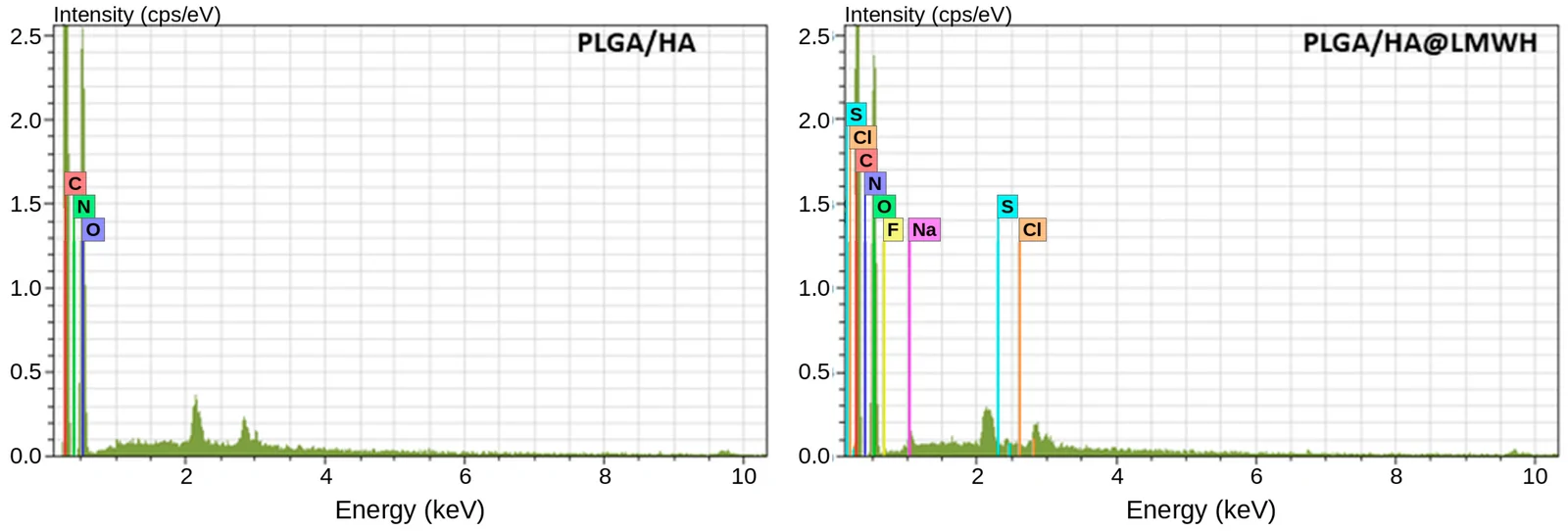
EDX patterns of PLGA/HA and PLGA/HA@LMWH nanofiber membranes.

Furthermore, EDX elemental analysis was performed to verify heparin loading (Figure 5). The appearance of a distinct sulfur peak (0.72% S) in PLGA/HA@LMWH, which was entirely absent in PLGA/HA, provided direct evidence that LMWH was successfully integrated into the PLGA/HA scaffold.

**Figure 6:**
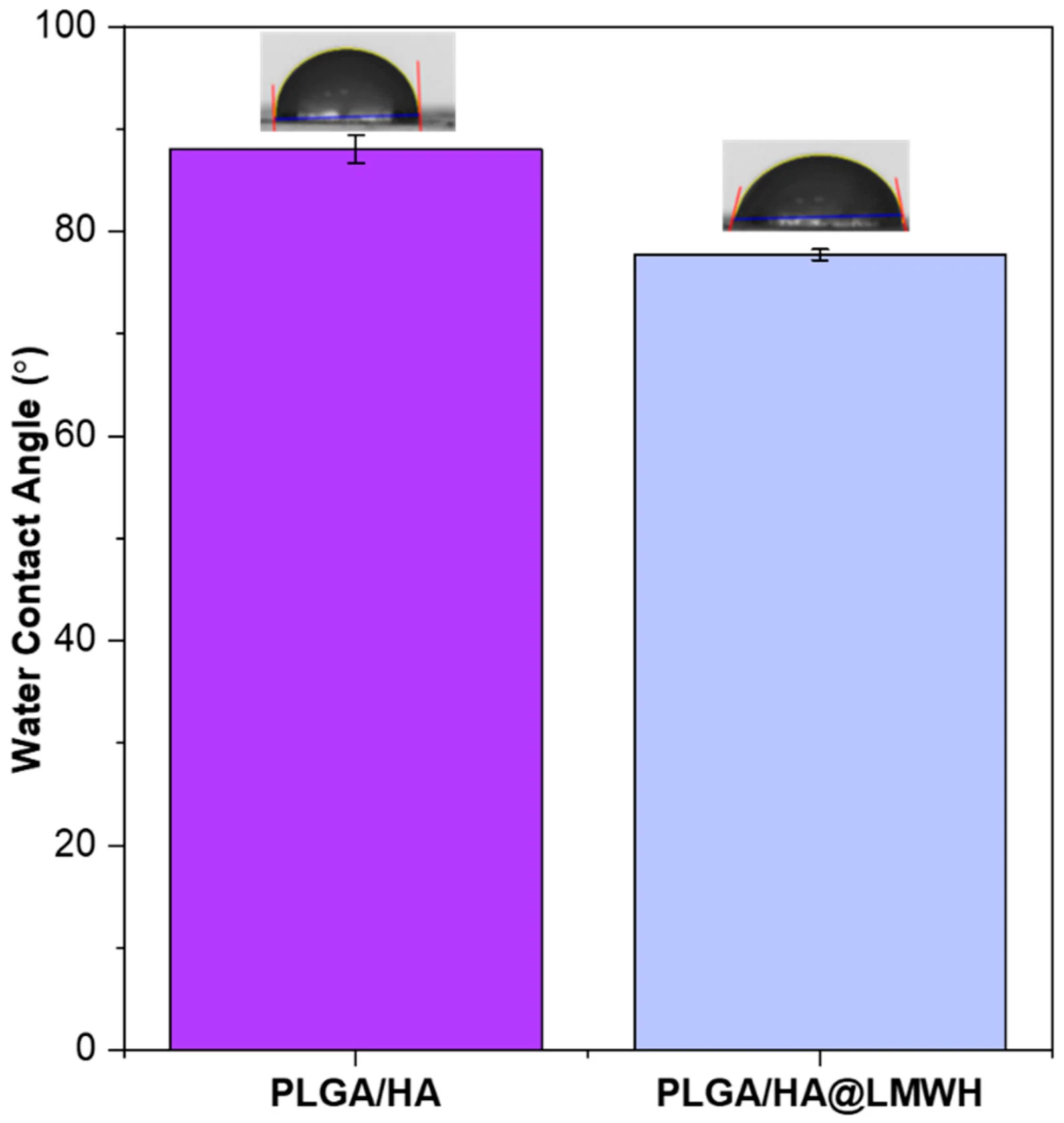
Water contact angles of PLGA/HA and PLGA/HA@LMWH nanofibers. The red lines indicate the baseline and tangent lines used for contact angle determination.

Figure 6 shows water contact angles of PLGA/HA and PLGA/HA@LMWH nanofibers. It was found that the water contact angles of the PLGA/HA and PLGA/HA@LMWH nanofibers changed from ∼88° to ∼78°, respectively. In the literature, the contact angle of pure PLGA was found to be ≥90°, due to its hydrophobic nature. For instance, Zhang et al. reported that the contact angle of PLGA was 98° [36]. In another work, Zhang et al. reported that pristine PLGA showed 95.6° ± 6.2° [37]. However, the addition of hydrophilic HA increased surface wettability and reduced the water contact angle. Similarly, Zhang et al. [36] reported that HA decreased the water contact angle of pure PLGA. Likewise, the addition of LMWH reduced the water contact angle [36]. It was clearly observed that this change stemmed from the hydrophilic nature of heparin. For example, Wang et al. immobilized heparin on the PLGA surface and reported that the water contact angle decreased after further modification with heparin [38]. More strikingly, Ghamkhari et al. reported that PLGA had a water contact angle of 70.3°, which significantly decreased upon the conjugation of heparin to 7.2° due to the hydrophilicity of heparin [39].

#### 2.1.2. Results of In Vitro Heparin Release

**Figure 7:**
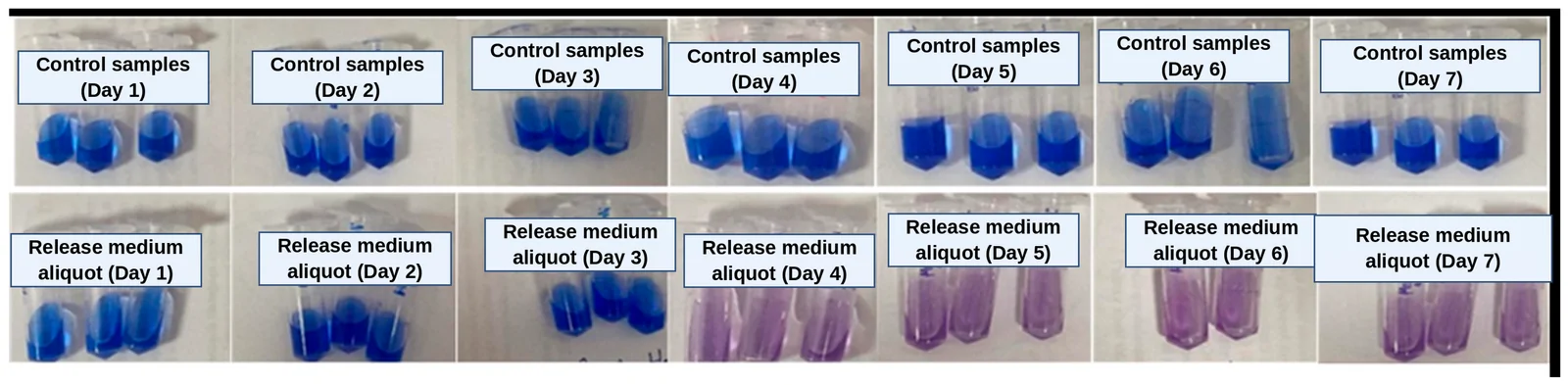
Colorimetric evaluation of LMWH release from PLGA/HA@LMWH nanofiber membranes over time using TB.

Figure 7 presents the colorimetric evaluation of LMWH release from PLGA/HA@LMWH nanofibrous membranes over time. As clearly shown in Figure 7, the presence of LMWH in the PLGA/HA@LMWH nanofibrous membranes and its release from these membranes were qualitatively evaluated over a 7-day period using the toluidine blue (TB) assay. A visual color change from dark blue to purple was observed over time, indicating the sustained presence of active heparin released into the medium and confirming the qualitative release trend. Control samples and samples from the release medium were collected daily (from day 1 to day 7). The gradual change in color intensity indicated sustained LMWH release. In particular, partial degradation of the nanofibrous membrane became evident from day 3 onward, accompanied by a distinct color change from dark blue to purple, indicating increased LMWH release.

**Figure 8:**
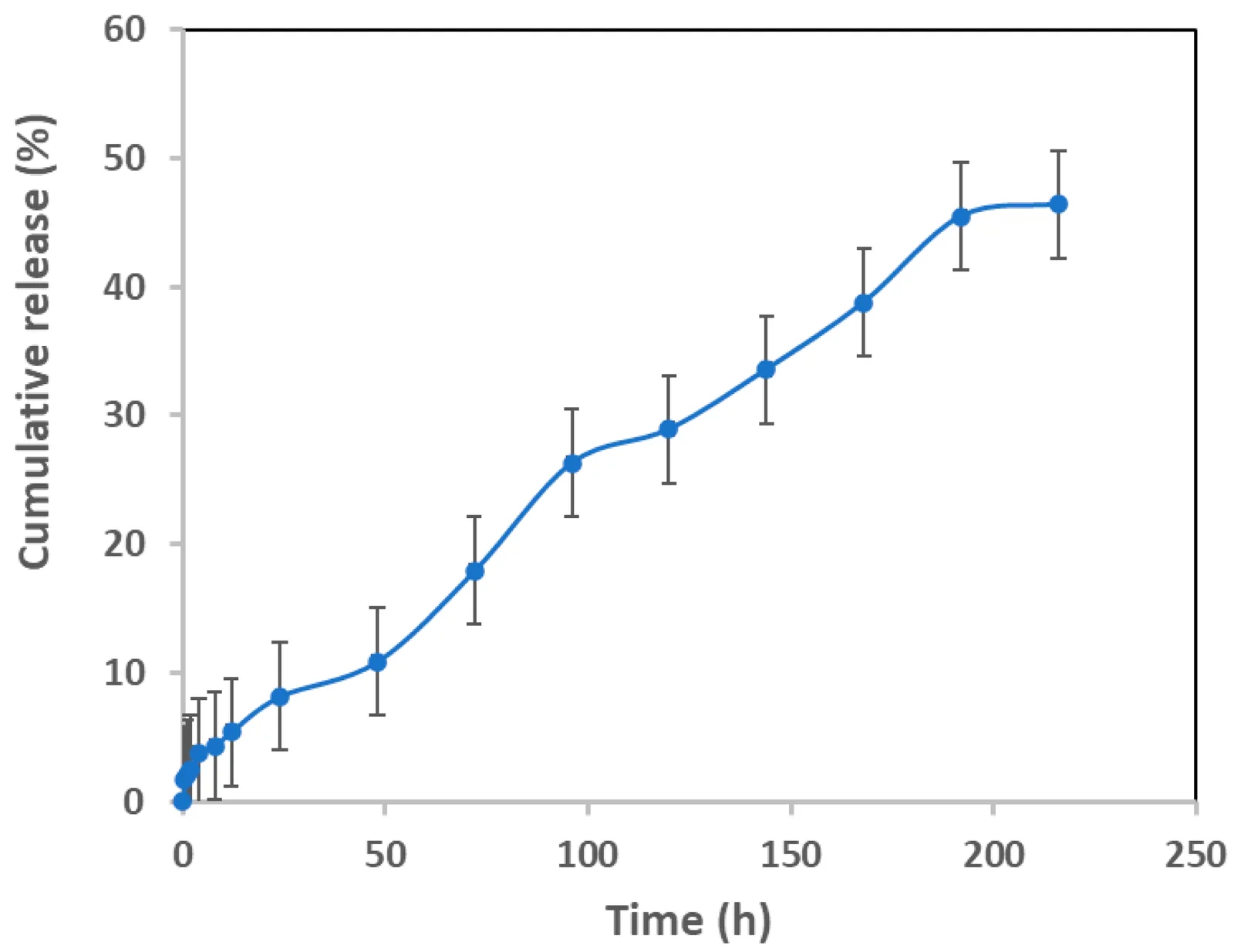
Cumulative release profile of LMWH from PLGA/HA@LMWH.

The cumulative in vitro release profile for LMWH is shown in Figure 8. As shown in Figure 8, the PLGA/HA@LMWH nanofiber membranes exhibited stable and sustained release profiles in PBS. Cumulative LMWH release increased gradually over the 216-h observation period, demonstrating that the PLGA/HA-based scaffold allowed for controlled LMWH release. No burst release was observed initially. The release amounts at 24 h, 48 h, and 72 h were 8.17%, 10.87%, and 17.92%, respectively. By 216 h, the release amount stabilized, reaching 46.4%. Intermolecular interactions between PLGA/HA and LMWH during the electrospinning process may have contributed to the formation of a relatively stable fibrous structure, thereby restricting the rapid diffusion of LMWH from the inner layer into the PBS medium and facilitating a slow, sustained release [40]. Taken together, although LMWH is inherently unstable and prone to degradation, these findings highlight the advantages of PLGA/HA as a drug carrier for sustained LMWH release and demonstrate that the material effectively minimizes burst release.

#### 2.1.3. Results of the Cell Viability Assessment

**Figure 9:**
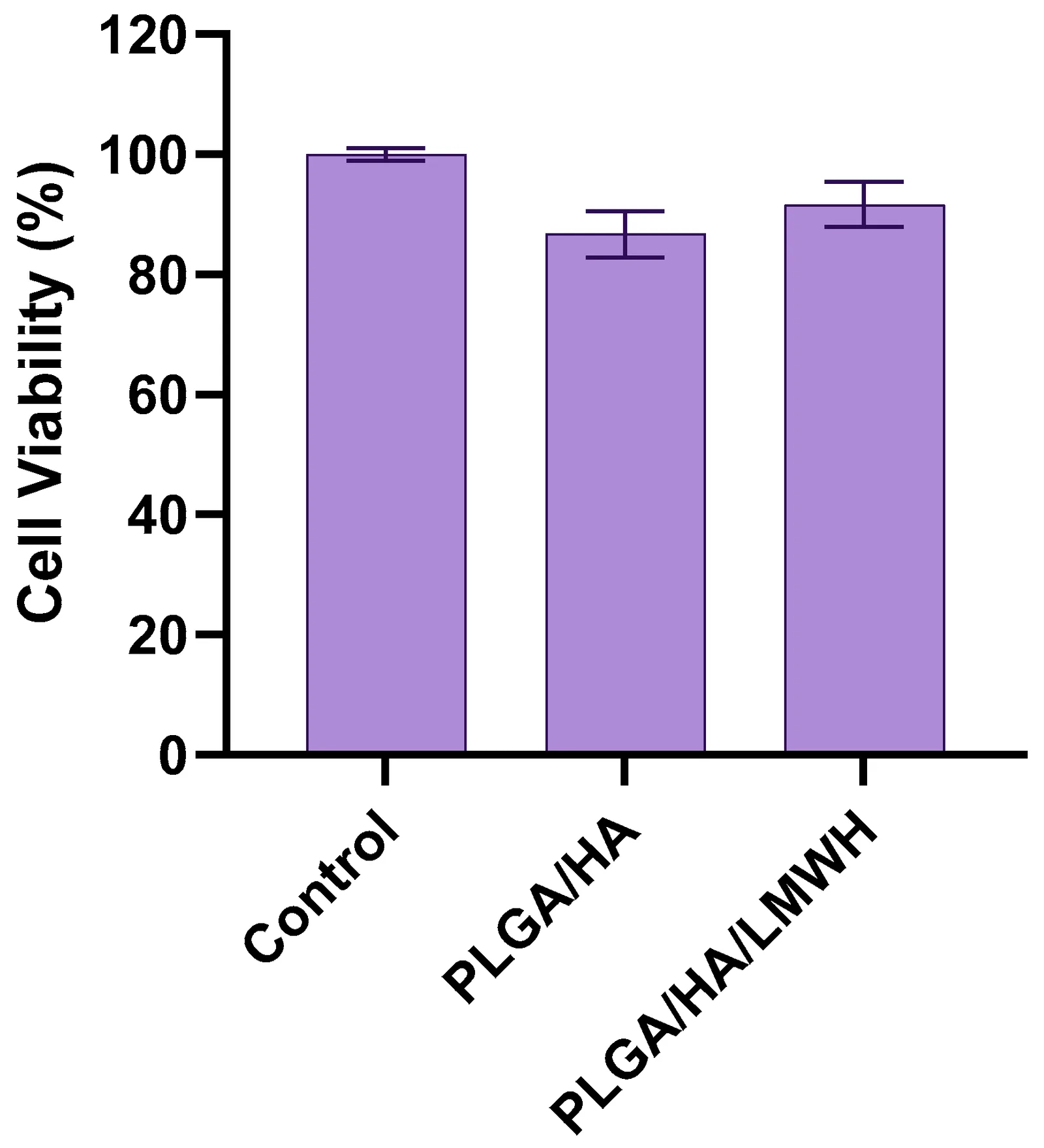
Cell viability of PLGA/HA and PLGA/HA@LMWH nanofibers.

**Figure 10:**
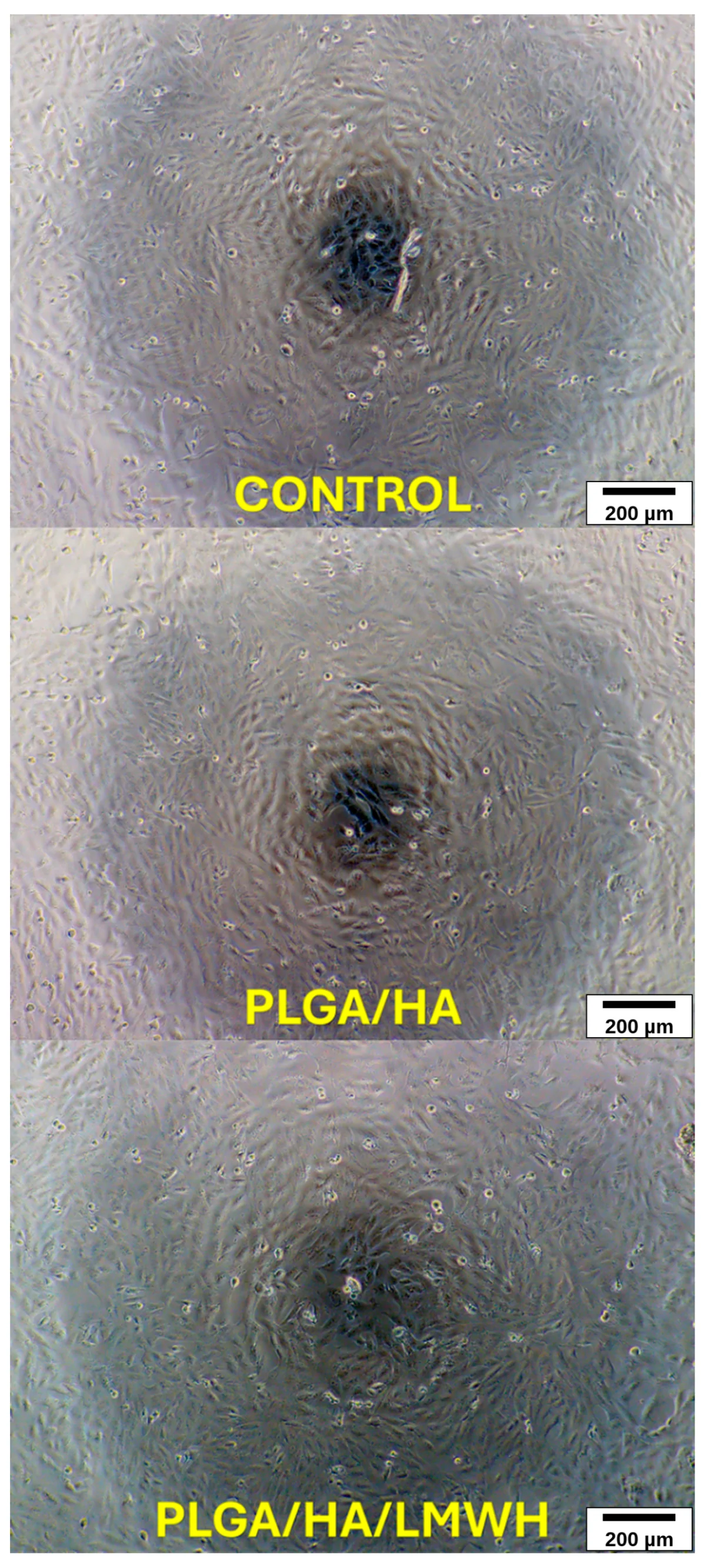
Optical microscope images of L929 cells after 24 h.

According to MTT test results, PLGA/HA (86.72% ± 3.83) and PLGA/HA@LMWH (91.67% ± 3.73) scaffolds exhibited high cell viability (Figure 9). These rates, which exceed the 70% threshold value specified in the ISO 10993-5 standard [41], demonstrate that both materials are non-cytotoxic and exhibit good biocompatibility [42]. Previous studies have also reported that PLGA and HA materials possess high biocompatibility and support high cell viability (>80–90%) [43]. Cell viability remained virtually unchanged following the incorporation of LMWH. Furthermore, optical microscope images in Figure 10 confirmed that, after a 24-h culture period, the cells were homogeneously distributed, adhered strongly to the surface, and maintained their natural morphology. These findings, which indicate an absence of cytotoxic effects, demonstrated that PLGA/HA@LMWH scaffolds serve as a suitable matrix for tissue engineering applications.

### 2.2. Results of Macroscopic Adhesion Scoring

**Figure 11:**
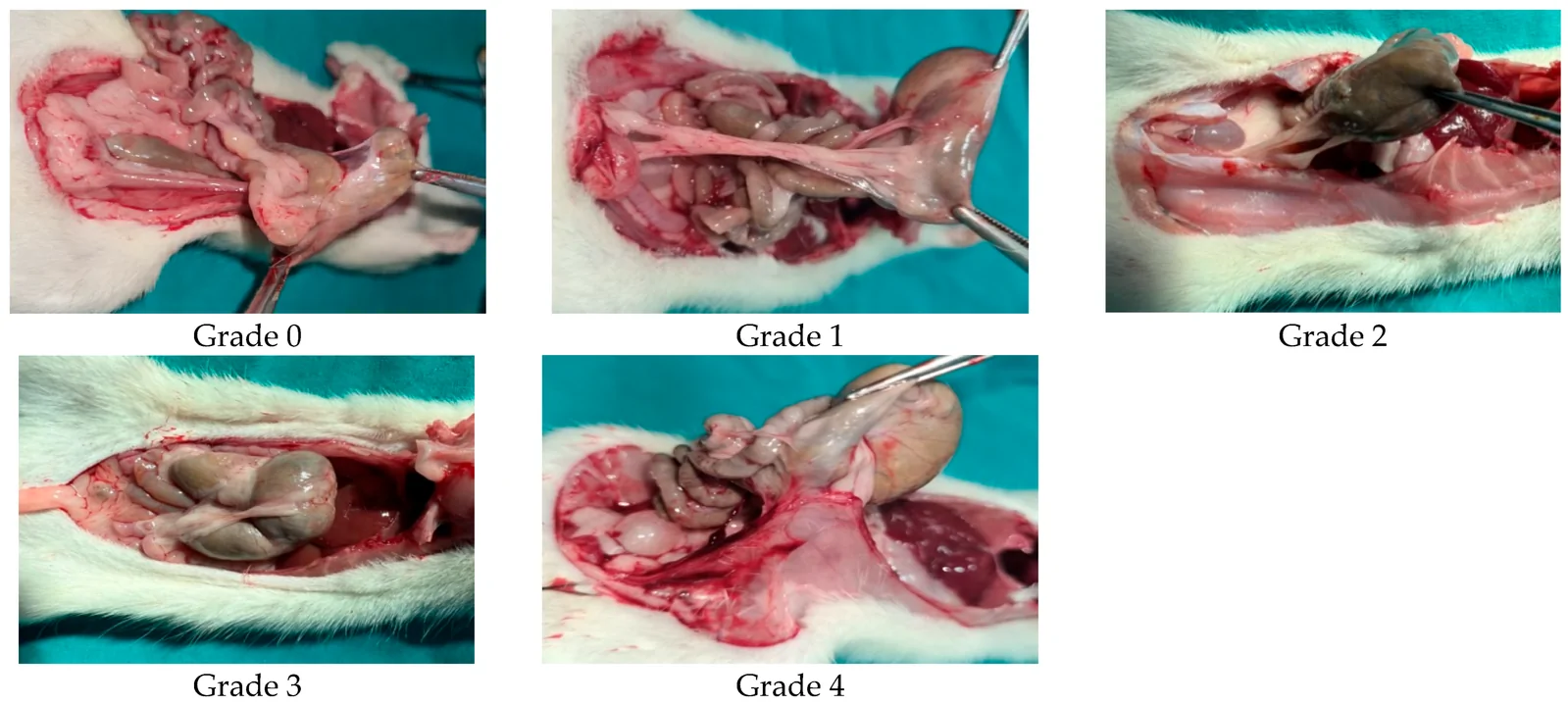
Images of intraabdominal adhesions in our study (according to the classification described by Nair et al. [44]).

No morbidity or mortality was observed during the study; rats were sacrificed on day 21. Macroscopic adhesions in rats with opened abdominal regions were evaluated by an independent surgeon according to the classification of Nair et al. [44] (Figure 11).

**Figure 12:**
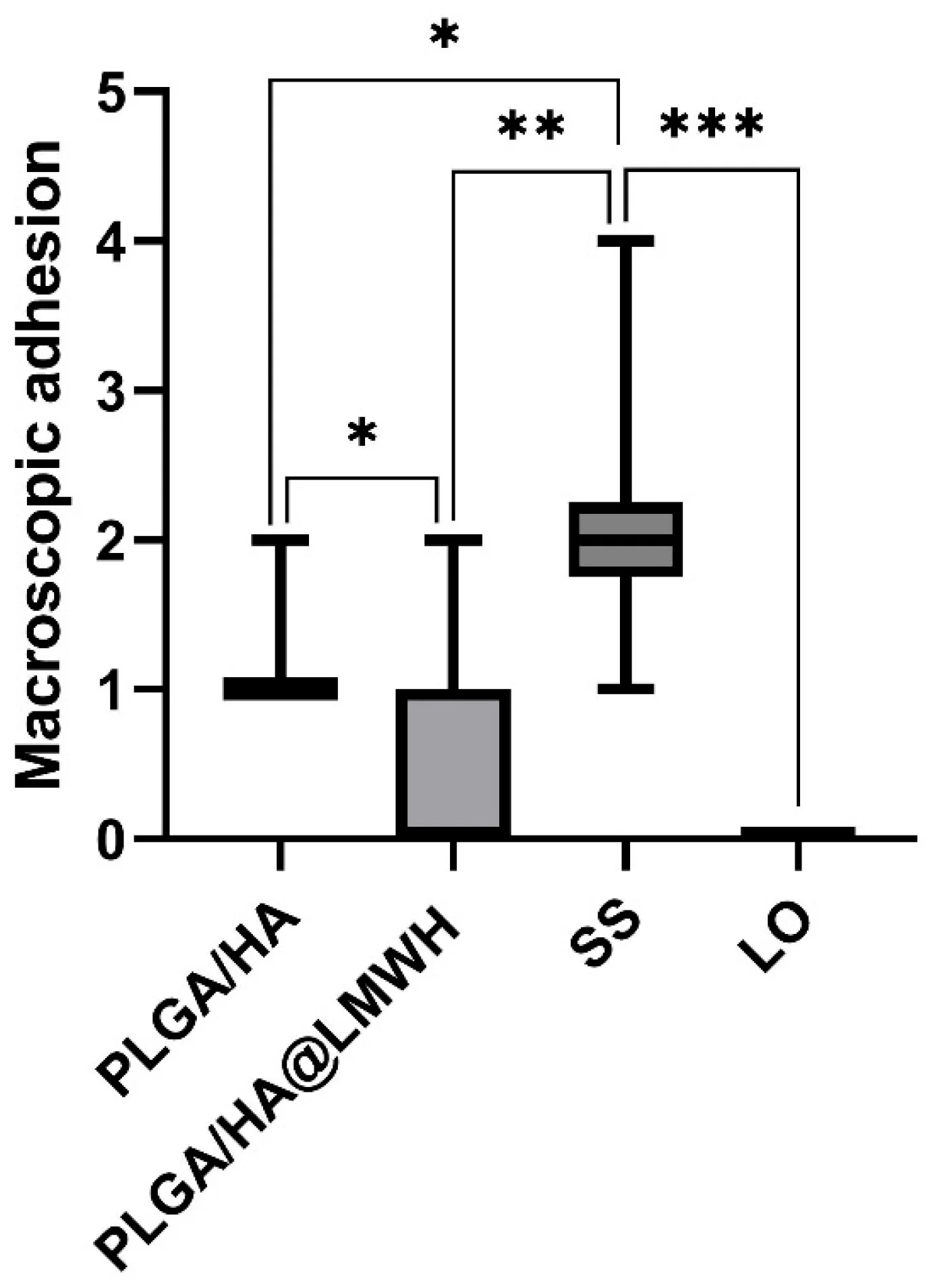
Macroscopic adhesion scores of the groups. Because the data did not show a normal distribution, the Kruskal–Wallis test was used to determine differences between groups, and Dunn’s multiple comparison test was used for pairwise comparisons. Data are expressed as median (IQR). Boxes represent the IQR, horizontal lines inside boxes indicate the median, and whiskers denote the range (*n* = 10 per group). *, **, *** mean statistically significant (*p* < 0.05), highly significant (*p* < 0.01), and extremely significant (*p* < 0.001), respectively. (SS: Saline Solution and LO: Laparotomy Only).

According to Figure 12, macroscopic adhesion scores were highest in the SS (Saline Solution) group. The scores of the PLGA/HA@LMWH group were significantly lower than those of the PLGA/HA and SS groups (*p* < 0.05, *p* < 0.01). The SS group exhibited significantly higher adhesion compared to PLGA/HA (*p* < 0.01) and LO (Laparotomy Only) (*p* < 0.001).

### 2.3. Results of Histopathological Evaluation

**Figure 13:**
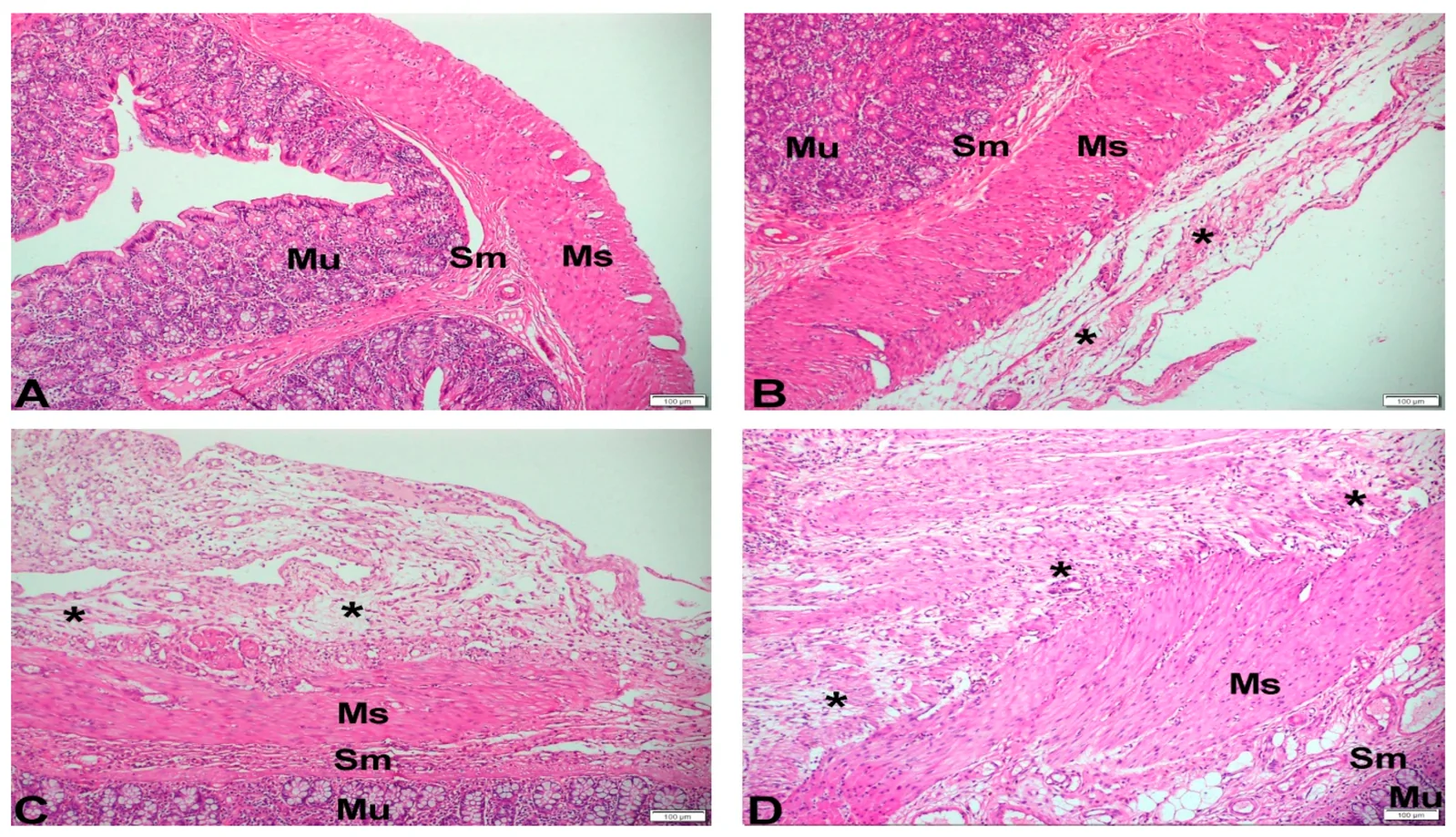
Fibrosis, neovascularization, and inflammatory cell infiltrations in the serosa of the cases (Mu: mucosa; Sm: submucosa; Ms: muscularis layer; *: adhesion area). (**A**) LO group; (**B**) PLGA/HA group; (**C**) PLGA/HA@LMWH group; (**D**) SS group.

Histopathological examination revealed a normal serosa in the LO group, while the PLGA/HA, PLGA/HA@LMWH, and SS groups showed adhesions characterized by varying degrees of inflammatory cell infiltration, fibrosis, and neovascularization (Figure 13).

**Figure 14:**
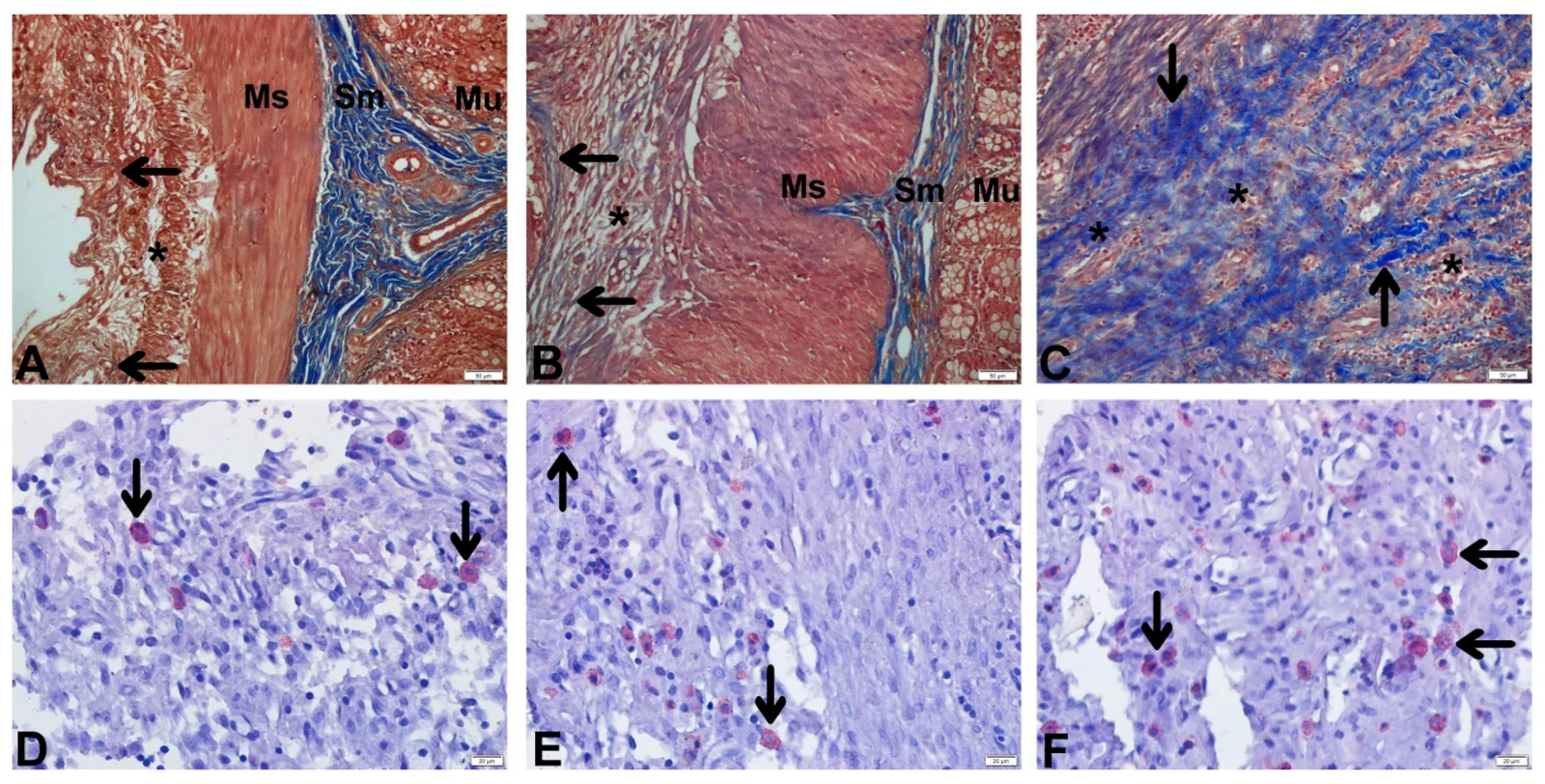
In the adhesion area, the arrows in (**A**–**C**) show collagen fibers and the arrows in (**D**–**F**) show intracytoplasmically positively stained cells. (Mu: mucosa; Sm: submucosa; Ms: muscularis layer; *: adhesion area).

Staining with Masson trichrome revealed collagen fibers stained blue to varying degrees in the adhesion area (Figure 14A–C). In some cases, they appeared as a small number of independent fibers (Figure 14A), while in others they were fused together (Figure 14B) or formed bundles (Figure 14C).

Immunohistochemical staining with TGF beta 1 (Transforming Growth Factor beta 1) monoclonal antibodies revealed intracytoplasmically positive stained cells in the adhesion area (Figure 14D–F).

**Figure 15:**
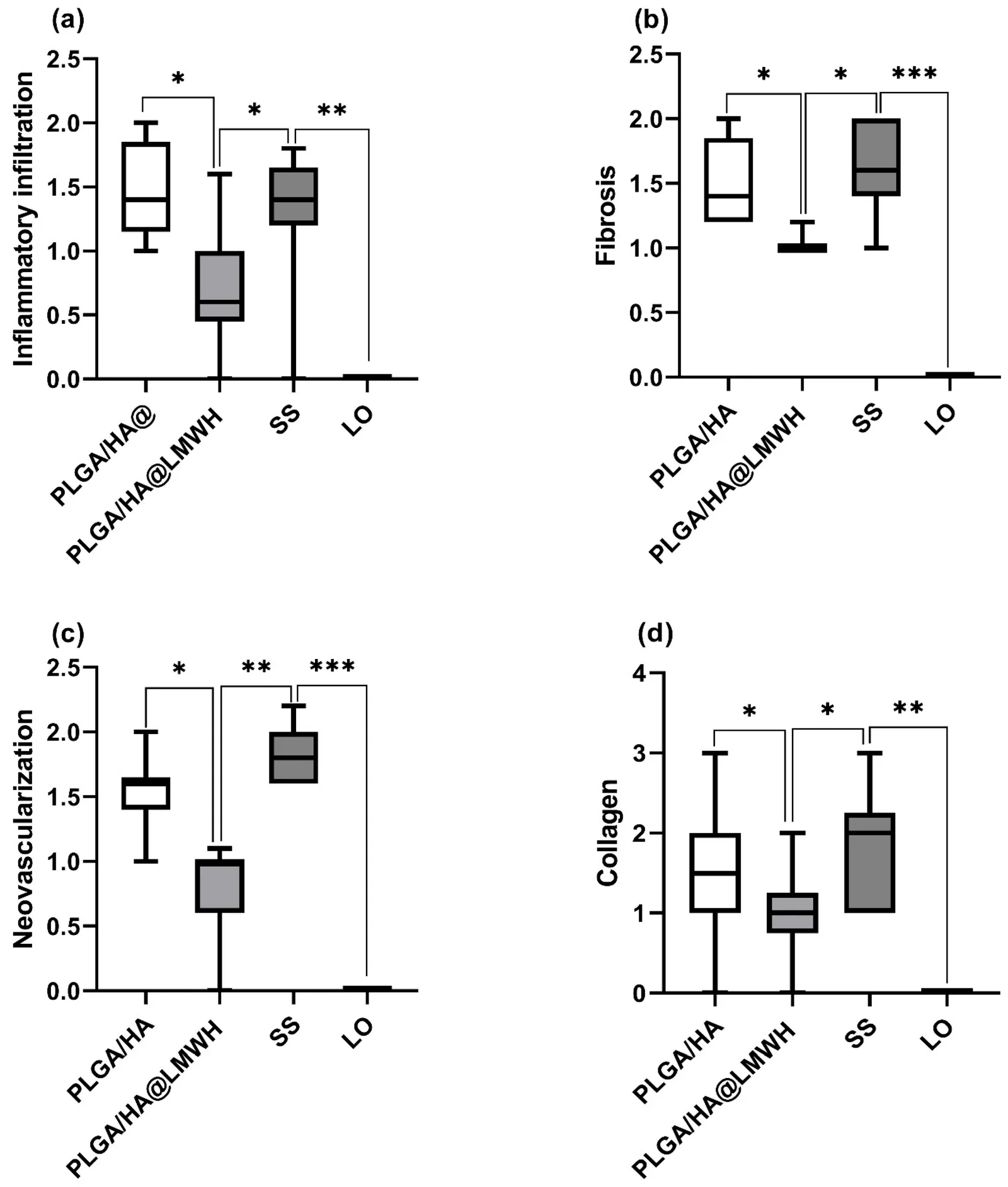
Comparison of histopathological findings. (**a**) The group comparison of inflamatory infiltration results, (**b**) The group comparison of fibrosis results, (**c**) The group comparison of neovascularization results, (**d**) The group comparison of collagen results. Because the data did not show a normal distribution, the Kruskal–Wallis test was used to determine differences between groups, and Dunn’s multiple comparison test was used for pairwise comparisons. Data are expressed as median (IQR). Boxes represent the IQR, horizontal lines inside boxes indicate the median, and whiskers denote the range (*n* = 10 per group). *, **, *** mean statistically significant (*p* < 0.05), highly significant (*p* < 0.01), and extremely significant (*p* < 0.001), respectively. SS: Saline Solution and LO: Laparotomy Only.

The highest inflammatory infiltration (Figure 15a) and fibrosis (Figure 15b) scores were observed in the PLGA/HA and SS groups. In the PLGA/HA@LMWH group, both parameters were significantly lower compared to PLGA/HA and SS (*p* < 0.05). The difference between PLGA/HA and LO was more significant in inflammation (*p* < 0.01), while the difference between SS and LO was more significant in fibrosis (*p* < 0.001). Neovascularization (Figure 15c) and collagen deposition (Figure 15d) were most pronounced in the SS and PLGA/HA groups. In the PLGA/HA@LMWH group, both parameters were significantly lower compared to PLGA/HA (*p* < 0.05) and SS (*p* < 0.01). The difference between SS and LO in neovascularization is highly significant (*p* < 0.001); collagen deposition is significantly higher in the SS group compared to LO (*p* < 0.01). These findings confirm heparin’s suppressive effect on inflammatory infiltration, fibrosis, neovascularization, and collagen deposition.

### 2.4. Results of Immunohistochemical Evaluation

**Figure 16:**
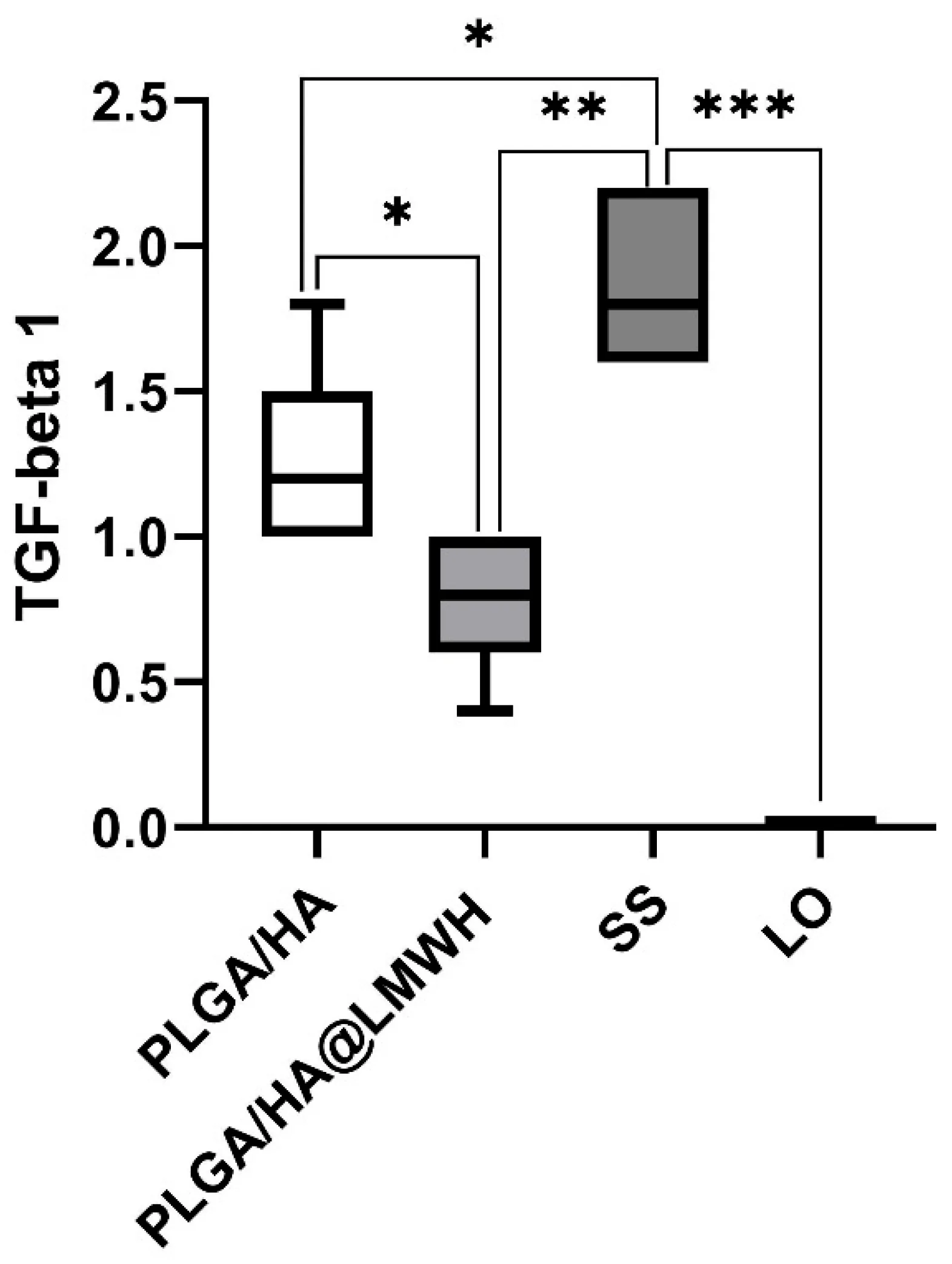
Comparison of TGF-β1 levels. Because the data did not show a normal distribution, the Kruskal–Wallis test was used to determine differences between groups, and Dunn’s multiple comparison test was used for pairwise comparisons. Data are expressed as median (IQR). Boxes represent the IQR, horizontal lines inside boxes indicate the median, and whiskers denote the range (*n* = 10 per group). *, **, *** mean statistically significant (*p* < 0.05), highly significant (*p* < 0.01), and extremely significant (*p* < 0.001), respectively. SS: Saline Solution and LO: Laparotomy Only.

Intracytoplasmic positivity was observed in cells in the adhesion areas in TGF-β1 monoclonal antibody staining (Figure 14D–F). According to the TGF-β1 analysis in Figure 16, the highest expression was observed in the SS group, followed by the PLGA/HA group. In the PLGA/HA@LMWH group, a significant decrease was detected compared to PLGA/HA (*p* < 0.05) and SS (*p* < 0.01); the difference between SS and LO was highly significant (*p* < 0.001). These findings also confirm the TGF-β1 suppressive effect of heparin.

### 2.5. Biochemical Evaluations

**Figure 17:**
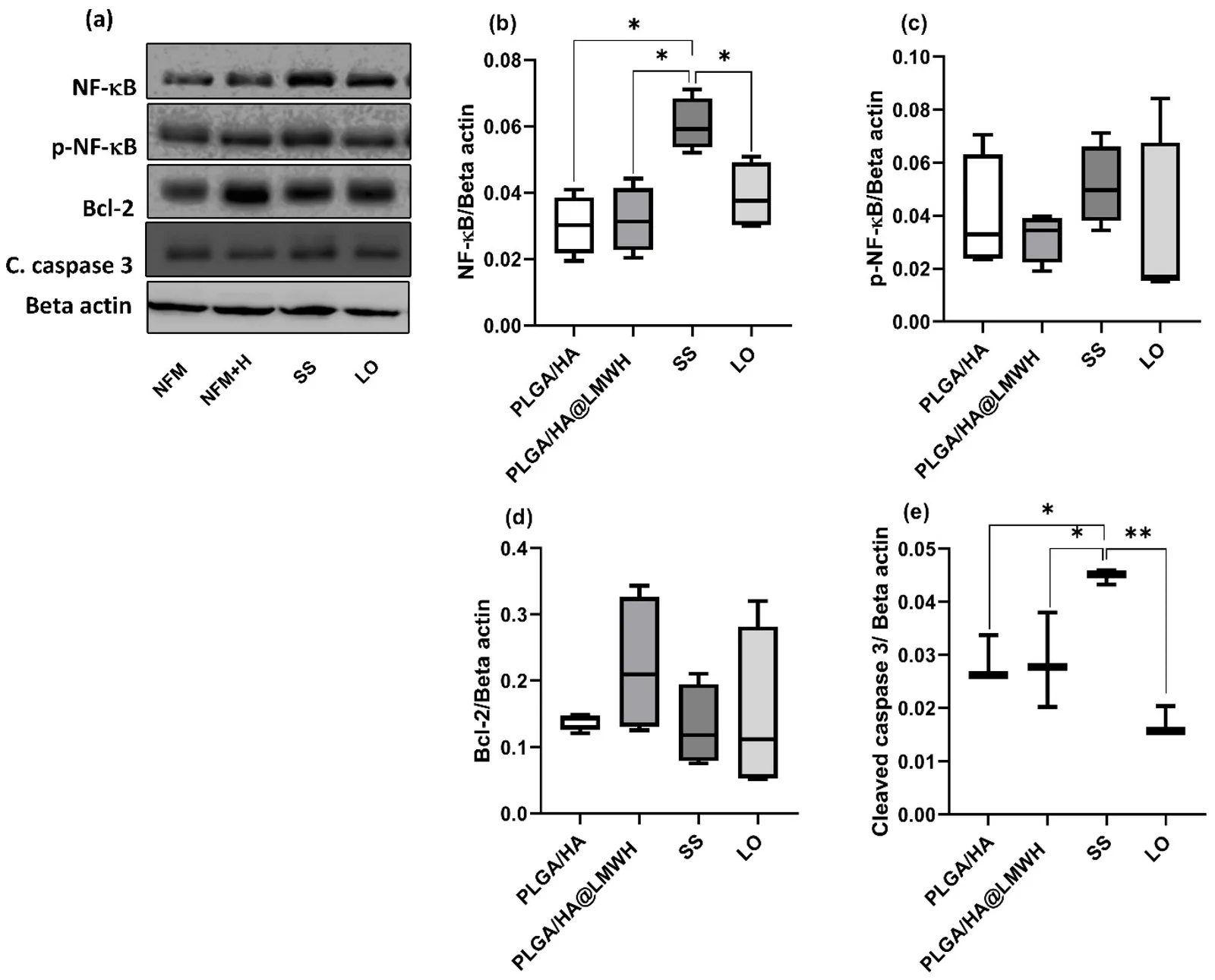
Western blot analysis results of NF-κB, p-NF-κB, Bcl-2, and cleaved caspase-3. (**a**) NF-κB, p-NF-κB, Bcl-2, and cleaved caspase-3 protein bands analyzed by Western blot. (**b**–**e**) The relative expression levels and statistical analysis of NF-κB, p-NF-κB, Bcl-2, and cleaved caspase-3 proteins. All experiments were independently repeated at least three times (*n* = 3 per group). The Kruskal–Wallis test was used to determine differences between groups, and Dunn’s multiple comparison test was used for pairwise comparisons. Data are expressed as median (IQR). Boxes represent the IQR, horizontal lines inside boxes indicate the median, and whiskers denote the range. *, ** mean statistically significant (*p* < 0.05), highly significant (*p* < 0.01), respectively. SS: Saline Solution and LO: Laparotomy Only.

On the 21st day after abrasion, tissue samples taken from PLGA/HA, PLGA/HA@LMWH, SS, and LO group rats were analyzed for NF-κB, p-NF-κB, Bcl-2, and cleaved caspase-3 levels using Western blot. According to Western blot analysis (Figure 17), increased NF-κB and cleaved caspase-3 levels in the SS group were significantly decreased in the PLGA/HA and PLGA/HA@LMWH groups (*p* < 0.05). In the PLGA/HA@LMWH group, Bcl-2 levels increased while cleaved caspase-3 levels decreased compared to the SS group (Figure 17d,e). Despite the observed alterations in p-NF-κB and Bcl-2 levels in both groups, the differences did not reach statistical significance.

These findings indicate that heparin inhibits cell death by suppressing the apoptotic pathway. A decrease in NF-κB indicates a transition to the resolution phase of inflammation, a decrease in cleaved caspase-3 suggests a slowing of cell death and tissue stabilization, while an increase in Bcl-2 indicates support for cell survival and successful continuation of tissue repair.

Western blot analysis of NF-κB, p-NF-κB, Bcl-2, and beta-actin (Figure 17a); relative expression levels of NF-κB, p-NF-κB, Bcl-2, and cleaved caspase-3 proteins and statistical analysis (*n* = 3 per group) (Figure 17b–e) are given in Figure 17.

**Figure 18:**
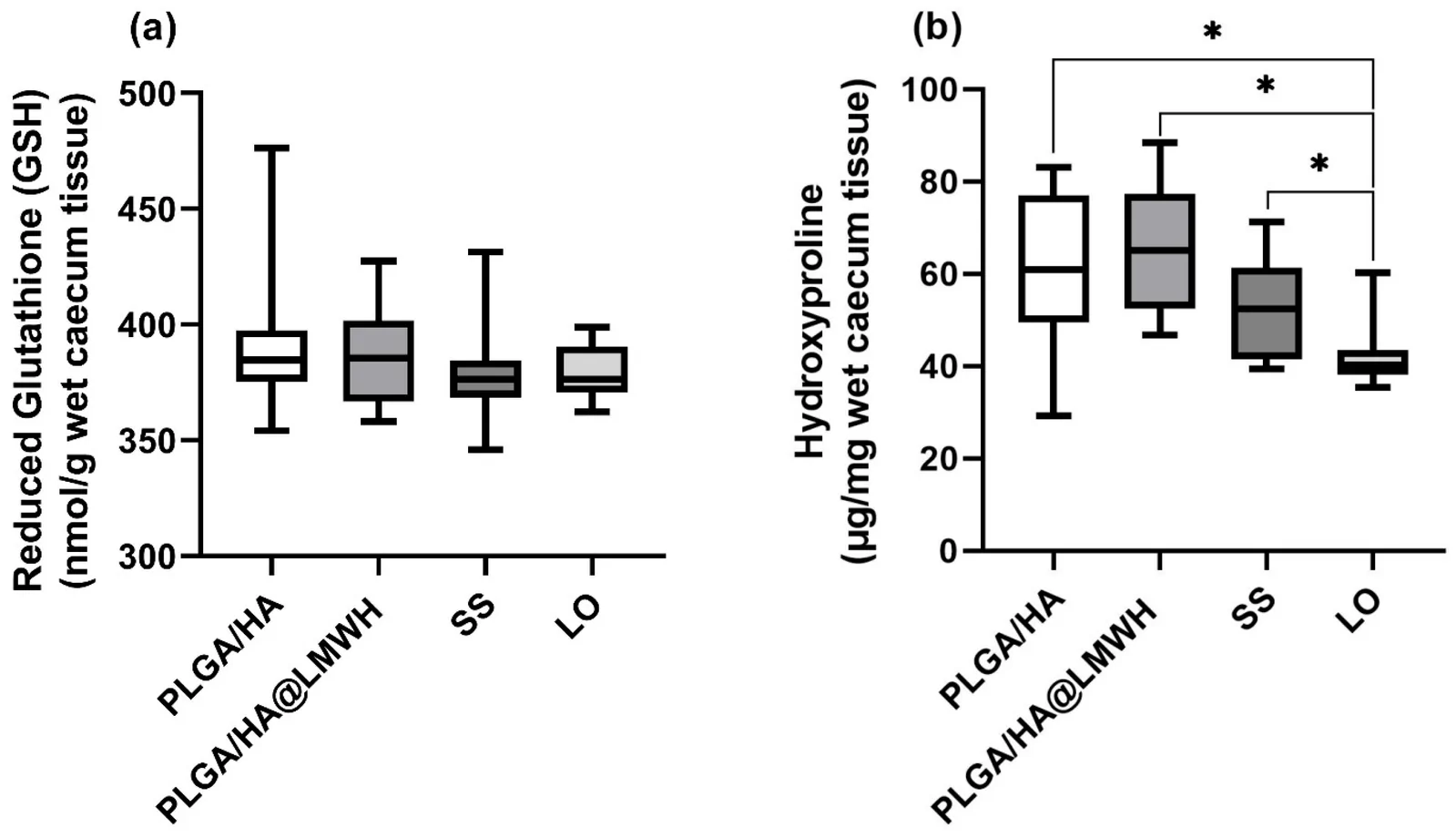
Comparison of glutathione and hydroxyproline values in the groups. (**a**) The group comparison of reduced glutathione results (**b**) The group comparison of hydroxyproline results, Because the data did not show a normal distribution, the Kruskal–Wallis test was used to determine differences between groups, and Dunn’s multiple comparison test was used for pairwise comparisons. Data are expressed as median (IQR). Boxes represent the IQR, horizontal lines inside boxes indicate the median, and whiskers denote the range; (*p* < 0.05) is considered statistically significant. * means statistically significant (*p* < 0.05) (*n* = 10 per group). SS: Saline Solution and LO: Laparotomy Only.

GSH levels were found to be statistically similar in all groups (Figure 18a). This can be attributed to the completion of wound healing and antioxidant system compensation on day 21. Hydroxyproline levels were found to be significantly higher (*p* < 0.05) in the PLGA/HA, PLGA/HA@LMWH, and SS groups compared to the LO group (Figure 18b); however, no statistically significant difference was observed in terms of fibrotic response among these three groups.

## 3. Discussion

Postoperative intraabdominal adhesions are a significant problem that reduces quality of life, developing with inflammation, fibrin deposition, and fibroblast proliferation. The ideal approach is to prevent fibrotic bands without disrupting physiological healing. Therefore, biocompatible and biodegradable barrier membranes are an effective strategy [4,45,46].

This study investigated the effectiveness of electrospinning-produced PLGA/HA nanofiber membranes and PLGA/HA@LMWH membranes obtained by adding low-molecular-weight heparin in preventing intraabdominal adhesions in a rat model. The findings showed that the PLGA/HA@LMWH combination was superior to other groups in terms of inflammation, fibrosis, neovascularization, collagen deposition, TGF-β1 expression, and apoptotic pathways.

### 3.1. Design and Characterization of Nanofiber Membranes

Electrospinning is widely used in tissue engineering by producing nanofiber structures with high surface area/volume ratio, controlled porosity and extracellular matrix (ECM)-like morphology [34,47]. In our study, the homogeneous morphology of nanofiber membranes produced with PLGA/HA was confirmed by SEM.

The mechanical strength of PLGA and the hydrophilic structure and biocompatibility of HA make the membrane ideal for adhesion barriers [33]. HA’s transient ECM function and modulation of inflammation contribute to the biological performance of PLGA/HA membranes [48,49].

In the PLGA/HA@LMWH spectrum, the characteristic bands of PLGA and HA are preserved, while the LMWH-specific bands at 1220–1260 and 1030–1080 cm^−1^ and the sulfate ester band at 840–850 cm^−1^ become more prominent. This situation, similar to previous studies, shows that LMWH was successfully integrated into the nanofiber matrix while maintaining its chemical structure during electrospinning [30,31,32]. With the addition of LMWH, the average fiber diameter increased from 290 ± 5 nm to 320 ± 5 nm, accompanied by a slight broadening of the distribution. It has also been reported in the literature that the incorporation of bioactive molecules can lead to a slight increase in fiber diameter [30,32]. The preservation of the homogeneous and bead-free morphology of the nanofibers despite the addition of heparin demonstrates the successful integration of LMWH into the polymeric matrix. Both membranes retained properties critical for the adhesion barrier, such as a high surface area/volume ratio and controlled porosity.

MTT analysis revealed that PLGA/HA and PLGA/HA@LMWH nanofibers showed high biocompatibility in L-929 fibroblast cells. The higher cell viability exhibited by PLGA/HA@LMWH indicates that LMWH supports early cell proliferation by enhancing cell adhesion, proliferation, and surface hydrophilicity. These findings support the idea that the PLGA/HA@LMWH scaffold could be a suitable matrix for tissue engineering applications.

### 3.2. Macroscopic Adhesion Evaluation

The findings obtained in this study demonstrated that the macroscopic adhesion scores of the PLGA/HA@LMWH group were significantly lower compared to the PLGA/HA and SS groups (*p* < 0.05$, *p* < 0.01). Furthermore, the SS group exhibited significantly higher adhesions compared to the PLGA/HA (*p* < 0.01) and LO (*p* < 0.001) groups. These results reveal that the developed PLGA/HA@LMWH membrane holds promising potential in the prevention of postoperative adhesions.

In the prevention of adhesion formation, two primary strategies stand out: creating a physical barrier and suppressing the inflammatory response. The PLGA/HA@LMWH membrane offers a multifunctional approach that combines these two strategies.

PLGA (poly(lactic-co-glycolic acid)) is an FDA-approved polymer widely used in tissue engineering due to its biocompatibility and tunable degradation properties. HA (hyaluronic acid), on the other hand, is a glycosaminoglycan known for supporting wound healing and possessing potential anti-adhesion properties. The PLGA/HA combination provides tissue separation by forming a temporary physical barrier at the surgical site and mechanically reduces adhesion formation by preventing fibrin deposition. This physical barrier effect is supported by the significantly lower adhesion scores exhibited by the PLGA/HA group compared to the SS group.

However, it is emphasized that physical barriers alone may remain insufficient, and anti-inflammatory activity is also necessary, particularly in cases where the inflammatory response is intense. Indeed, the literature shows that PLGA-based membranes containing anti-inflammatory agents such as ibuprofen, flurbiprofen, chondroitin sulfate, or oregano essential oil are more effective in preventing adhesions.

Postoperative fibrin clots become permanent if the fibrinolytic system is suppressed, creating a matrix for fibrosis [50]. The anti-adhesive effect of heparin relies on its anticoagulant and anti-inflammatory properties; by binding to antithrombin III, it suppresses inflammatory cell adhesion, angiogenesis, and fibroblast proliferation while promoting fibrinolytic activity [12,51,52].

Although the anti-adhesive effect of heparin derivatives is known in the literature, studies utilizing local and controlled release strategies with nanofiber membranes are limited [27,53,54].

In our study, the loading of LMWH onto the nanofiber membrane provided locally controlled heparin release, minimizing systemic side effects. While the nanofiber membrane acted as a physical barrier, the LMWH-incorporated version possessed both physical and chemical barrier properties.

Here, the addition of LMWH to the PLGA/HA membrane provided a marked improvement in adhesion scores. In addition to its anticoagulant effect, the anti-inflammatory properties of LMWH are well-documented. Suppressing inflammation—which plays a critical role in adhesion formation—promotes the activation of the fibrinolytic system, inhibits fibrin deposition, and consequently reduces adhesion formation. The lowest adhesion scores observed in the PLGA/HA@LMWH group point to the additional benefit provided by LMWH through these mechanisms.

The findings obtained are consistent with similar studies in the literature. Jamshidi-Adegani and colleagues reported that ibuprofen-loaded PLGA nanofiber membranes significantly reduced adhesion bands in a mouse model [55]. Zhu et al. produced flurbiprofen axetil-loaded PVP/PLGA composite nanofibers by coaxial electrospinning [56]. It was determined that the drug-loaded membrane prevented adhesion and significantly reduced drug release. Mao et al. showed that sandwich-structured ibuprofen-loaded electrospun scaffolds effectively inhibited fibroblast adhesion and proliferation while providing long-term drug release [57]. Nadri et al. revealed that ibuprofen-loaded PEG/silk core–shell nanofiber membranes alleviated postoperative adhesions in a rat model [58]. Gao and colleagues demonstrated that PLGA electrospun membranes containing chondroitin sulfate provided anti-adhesion efficacy by increasing the M2-type macrophage ratio and reducing pro-inflammatory cytokines (IL-1β, TNF-α, IL-6) [59]. Wu et al. reported that PLGA membranes functionalized with lecithin and oregano essential oil suppressed oxidative stress and inflammation by regulating Nrf2/NF-κB signaling pathways and prevented adhesions [60]. These studies support the general trend that the incorporation of anti-inflammatory or bioactive molecules into PLGA-based membranes enhances adhesion prevention performance and align with the findings of the current study.

Additionally, in an experimental intraabdominal adhesion model conducted by Toyama and colleagues using the commercial product Seprafilm, macroscopic adhesion scores in the Seprafilm group were found to be remarkably lower compared to the control group (*p* < 0.01) [61]. Similar to the findings of Toyama et al. [61], the macroscopic adhesion scores in the PLGA/HA@LMWH group in our study were determined to be statistically significantly lower than those in the PLGA/HA group (*p* < 0.05) and remarkably lower than those in the SS group (*p* < 0.01).

The PLGA/HA@LMWH membrane developed in this study provides an effective approach in preventing postoperative abdominal adhesions by combining physical barrier function with the anti-inflammatory and anticoagulant effects of LMWH. The significantly lower adhesion scores observed in the PLGA/HA@LMWH group indicate that this multifunctional membrane is a promising candidate for clinical applications.

### 3.3. Evaluation of Histopathological Findings

Histopathological examinations showed that inflammation was highest in the PLGA/HA group, while it was significantly lower in the PLGA/HA@LMWH group (*p* < 0.05) (Figure 15a). This suggests that the nanofiber membrane may cause mild inflammation, but this response is suppressed by the addition of heparin. Acidic products formed during the degradation of PLGA may contribute to mild inflammation [62]. Heparin suppresses inflammation by inhibiting neutrophil adhesion, pro-inflammatory cytokine release, and inflammatory cell activation [63].

Fibrosis scores were found to be significantly higher in the SS and PLGA/HA groups compared to the PLGA/HA@LMWH group (*p* < 0.05). This can be explained by heparin inhibiting fibroblast proliferation and collagen synthesis, modulating factors such as transforming growth factor-beta (TGF-β) and fibroblast growth factor (FGF) [12,27,28]. Furthermore, the high fibrosis scores in the PLGA/HA group indicate that the physical barrier alone is insufficient to prevent fibrotic response and that pharmacological support is necessary.

Neovascularization was highest in the SS group, while it was significantly lower in the PLGA/HA@LMWH group (*p* < 0.01). Excessive vascularization during the adhesion process contributes to the persistence of adhesions. The effect of heparin on angiogenesis is biphasic; at low concentrations it can inhibit angiogenesis by modulating the activity of angiogenic factors (VEGF, bFGF), while at high concentrations it can exhibit a pro-angiogenic effect [27,64,65]. It is thought that the LMWH concentration used in our study suppressed neovascularization in the PLGA/HA@LMWH group by inhibiting angiogenesis.

Collagen accumulation was found to be high in the SS and PLGA/HA groups, while it was low in the PLGA/HA@LMWH group (*p* < 0.05). This finding indicates that heparin suppresses collagen synthesis through TGF-β1 inhibition or fibroblast modulation [28].

### 3.4. Immunohistochemical Findings: TGF-β1 Expression

In our study, TGF-β1 expression was highest in the SS group and significantly lower in the PLGA/HA@LMWH group (*p* < 0.01). This result shows that PLGA/HA@LMWH application inhibits adhesion formation by suppressing TGF-β1 signaling. TGF-β1 plays a central role in adhesion pathogenesis; it induces fibroblast proliferation, ECM synthesis, and myofibroblast differentiation [66]. The higher TGF-β1 in the PLGA/HA group compared to PLGA/HA@LMWH indicates that the maximum effect is achieved with the heparin combination.

### 3.5. Biochemical Findings: Inflammatory and Apoptotic Pathways

NF-κB and p-NF-κB levels were found to be significantly lower in the PLGA/HA and PLGA/HA@LMWH groups compared to the SS group (*p* < 0.05). Suppression of NF-κB activity indicates that the inflammatory response is controlled. NF-κB, activated after surgical trauma, plays a critical role in initiating the inflammatory cascade that triggers adhesion formation. NF-κB is central to the inflammatory response by regulating the expression of pro-inflammatory mediators [67,68].

In the PLGA/HA@LMWH group, anti-apoptotic Bcl-2 levels were significantly increased, while pro-apoptotic cleaved caspase-3 levels were decreased (*p* < 0.05). Bcl-2 supports cell survival by inhibiting the mitochondrial apoptotic pathway, while cleaved caspase-3 is an effector caspase that is activated in the final stages of apoptosis and makes cell death irreversible [69,70]. Post-surgical trauma, ischemia, inflammation, and oxidative stress induce apoptosis in peritoneal mesothelial cells, creating a predisposition to adhesion [71]. Suppression of apoptosis supports mesothelial regeneration. This, observed in the PLGA/HA@LMWH group, demonstrates that heparin protects tissue integrity by suppressing apoptotic cell death.

The similarity of GSH levels in all groups can be explained by the regression of the acute oxidative stress response on day 21. Although hydroxyproline levels were significantly higher (*p* < 0.05) in the PLGA/HA, PLGA/HA@LMWH, and SS groups compared to the LO group, no statistically significant difference was observed in terms of fibrotic response among these three groups. This indicates that a fibrotic response developed in all abrasion groups. Hydroxyproline is a specific amino acid found in the structure of collagen and is a marker of tissue collagen content [72]. While lower hydroxyproline levels were expected in the PLGA/HA@LMWH group, the lack of a statistically significant difference may be related to the sample size or the timing of the evaluation.

### 3.6. Clinical Applicability and Strengths of the Study

This study highlights the importance of a multidisciplinary approach in adhesion prevention. While the nanofibrillar membrane forms a physical barrier, controlled-release heparin exhibits local anti-inflammatory, anti-fibrotic, and anticoagulant effects. This synergistic approach provides more effective protection by targeting different stages of adhesion formation.

One of the key strengths of our study is the inclusion of a well-defined biomaterial design supported by comprehensive characterization studies. FT-IR and SEM analyses revealed the chemical structure and morphological features of the membranes in detail, demonstrating the success of heparin integration. Furthermore, the combined use of macroscopic, histopathological, immunohistochemical, and biochemical parameters in adhesion assessment allowed for a multidimensional interpretation of the results. In particular, the analysis of molecular markers such as NF-κB, p-NF-κB, Bcl-2, and cleaved caspase-3 provided important clues regarding the underlying mechanisms of adhesion pathogenesis.

### 3.7. Limitations of the Study and Future Perspectives

One limitation of this study is that the rat model may not fully reflect human surgical conditions. Peritoneal healing is faster in rats, and adhesion dynamics may differ.

Secondly, adhesion assessment was performed at a single time point (day 21). Since adhesion is a dynamic process, assessments at different time points may provide more detailed information.

Thirdly, the dose–response relationship of LMWH has not been investigated. The effectiveness of different heparin concentrations should be determined through optimization studies. In addition, in vivo characterization of membrane heparin release kinetics will provide a better understanding of the system’s performance.

Fourthly, a comparison of the anti-adhesive materials used in our study with commercial products (Seprafilm^®^, etc.) will be carried out in the next stage of this study; furthermore, the lack of such a comparison can be considered one of the limitations of our study.

Fifthly, the in vitro release of LMWH from nanofibrous membranes was qualitatively evaluated using colorimetric toluidine blue analysis. Additionally, a comprehensive time-dependent quantitative release profile was established.

Another limitation of this study is that only 3 independent repeated Western blot analyzes were performed on each group.

Future studies could investigate the efficacy of PLGA/HA@LMWH membranes in different adhesion patterns (ischemic, infectious). Combinations with other anti-adhesive agents may lead to more effective strategies.

## 4. Materials and Methods

### 4.1. Preliminary Study

#### Membrane Production and Characterization

**Figure 19:**
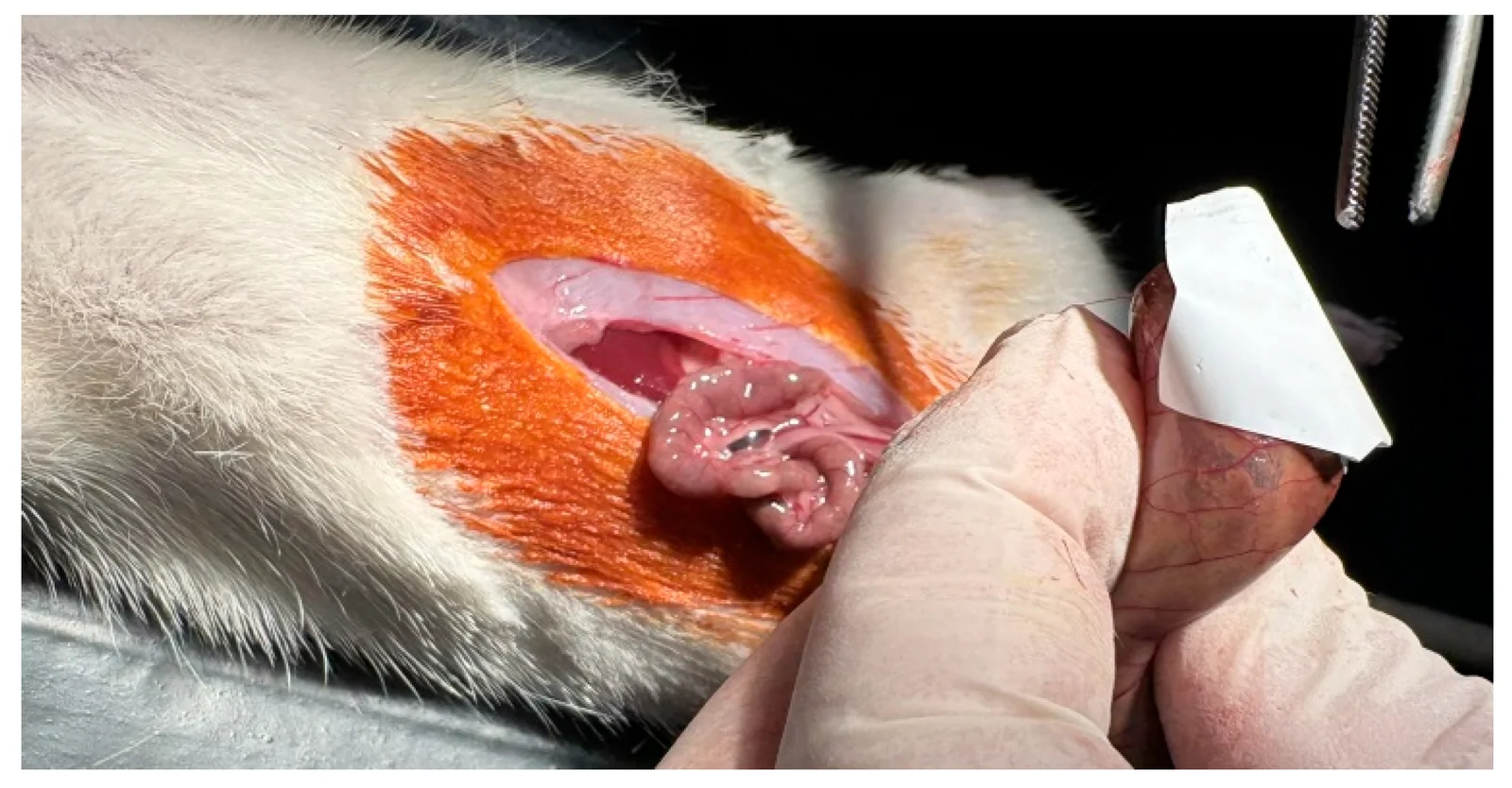
Application of nanofiber membrane to the abraded area in the cecum.

**Figure 20:**
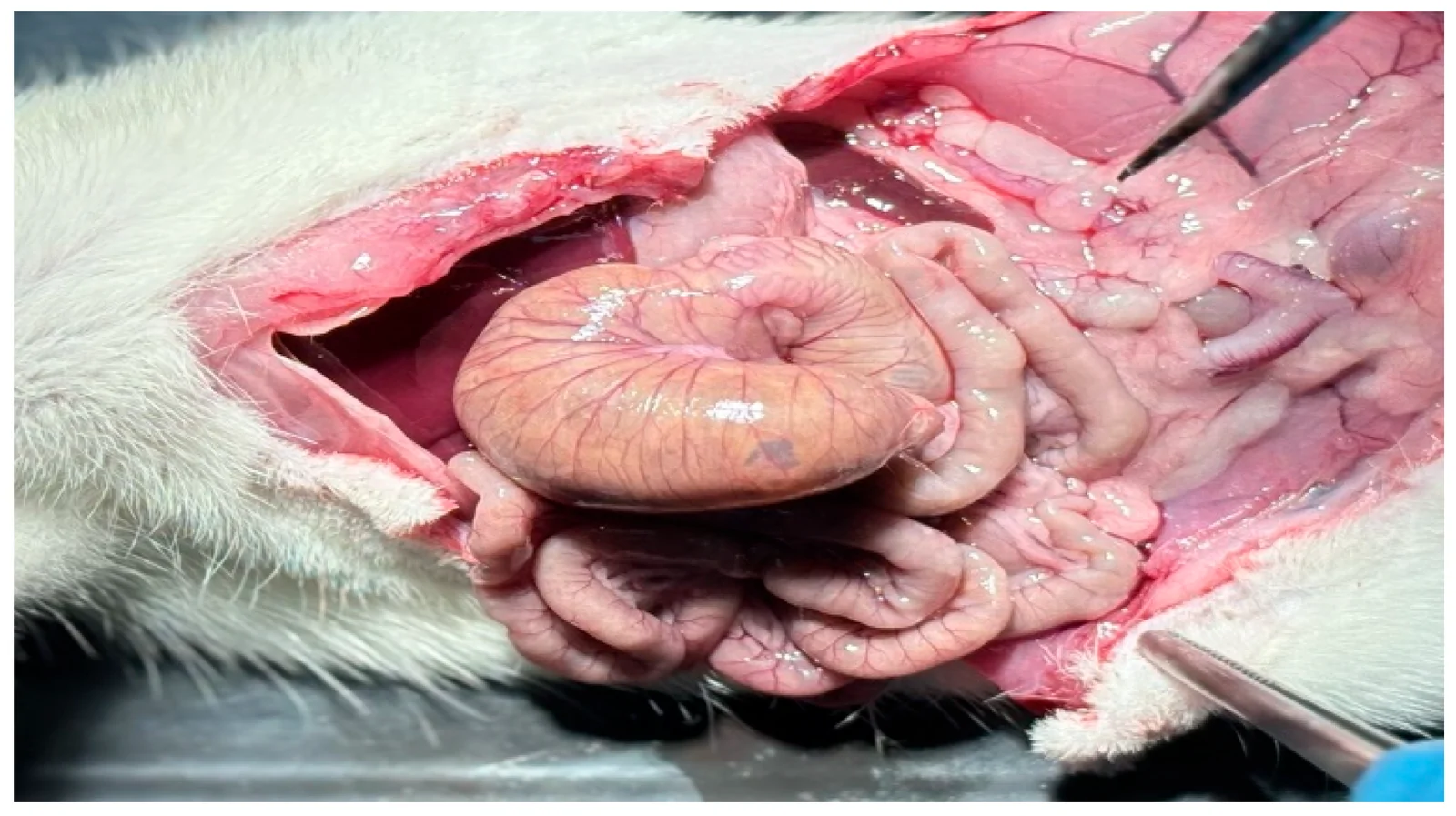
Intra-abdominal image 7 days after application of a nanofiber membrane.

**Figure 21:**
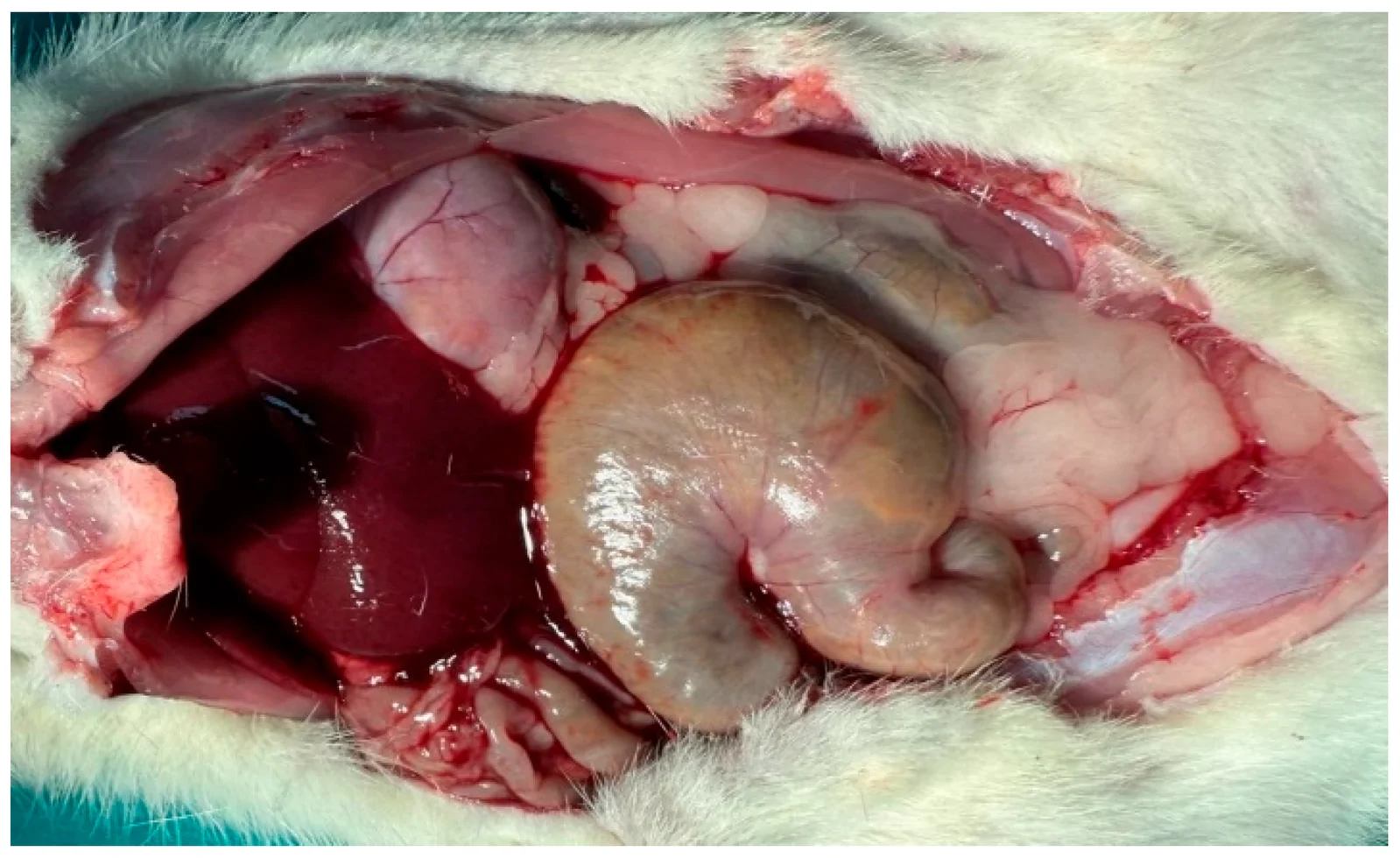
Intra-abdominal image 14 days after application of a nanofiber membrane.

In this study, biodegradable Poly-lactic-co-glycolic acid (PLGA) and Hyaluronic Acid (HA) polymers were used to produce nanofiber membranes by electrospinning. In optimization studies, in vitro experiments were conducted with at least two nanofiber membranes with different PLGA and HA formulation contents. Biocompatibility studies were investigated using L-929 mouse (*Mus musculus*) fibroblast cells. In in vitro experiments, a preliminary study was conducted on rats with intra-abdominal adhesions created using two different membranes that were tissue-compatible and biodegraded within one week. These two different membranes were tested on 18 Wistar Albino female rats with intra-abdominal adhesions. The nanofiber membrane used in our study demonstrated the ability to easily adhere to abrasive surfaces, and it also exhibited no fragility (Figure 19). Each membrane was applied to 9 rats; 3 rats from each group were sacrificed on days 7, 14, and 21. In this preliminary study, the in vivo antiadhesive efficacy and biodegradability of membranes were tested in a rat-induced intraabdominal adhesion model. Based on the results of the preliminary study, it was decided to use the PLGA/HA nanofiber membrane containing 0.6 g PLGA and 0.24 g HA, which had the best in vivo antiadhesive efficacy and biodegradability within one week, in the main study. Figure 20 and Figure 21 show images of the in vivo nanofiber membrane biodegradation in rat abdomens 7 and 14 days after application of a nanofiber membrane consisting of a combination of 0.6 g PLGA and 0.24 g HA. Macroscopic images taken after 7 and 14 days show that the nanofiber membranes have biodegraded and disappeared (Figure 20 and Figure 21).

### 4.2. Main Study

#### 4.2.1. Materials

All chemicals used in this study were of analytical purity and, unless otherwise indicated, were used without further purification. Poly(lactic-co-glycolic acid) (PLGA) (mean Mn: 25,000), hyaluronic acid sodium salt (high molecular weight, >1 MDa), and 1,1,1,3,3,3-hexafluoro-2-propanol (HFIP, ≥99%) were supplied by Sigma-Aldrich. Dimethylformamide (DMF, ≥99.8%) and chloroform (CHCl_3_, 99.8%) were sourced from ISOLAB (Wertheim, Germany). Low-molecular-weight heparin (LMWH) (Enoxaparin sodium, OKSAPAR 6000, anti-Xa IU/0.6 mL) was supplied by Koçak Farma İlaç ve Kimya Sanayi A.Ş., Tekirdağ, Türkiye).

#### 4.2.2. Production of PLGA/HA and PLGA/HA@LMWH Nanofiber Membranes

**Figure 22:**
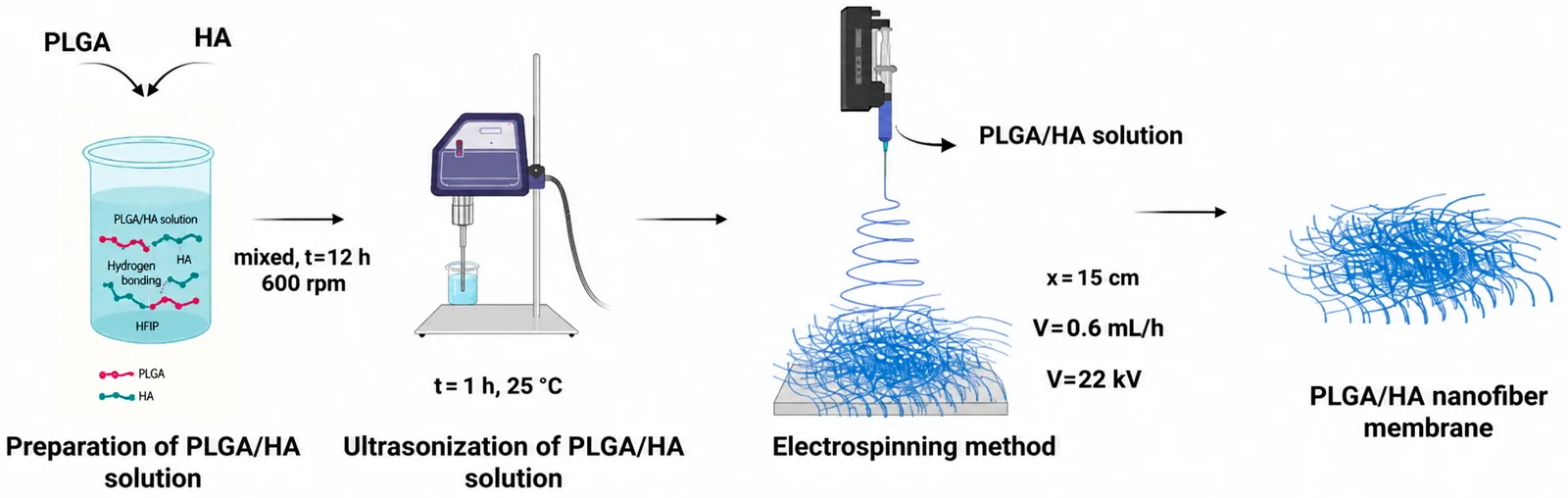
Preparation of PLGA/HA nanofiber membrane using electrospinning method.

In this study, biocompatible and biodegradable nanofiber membranes were developed to prevent postoperative adhesions. By adding LMWH to the polymer solution, membranes with dual-effect (chemical and physical) barrier properties were produced. In accordance with the literature, anti-adhesion PLGA/HA membranes with different porosities were produced by the electrospinning method [73]. For membrane fabrication, PLGA and HA (0.6 g/0.24 g) were dissolved in HFIP and mixed using a magnetic stirrer (Labron HS1-M3) at 600 rpm for 12 h at room temperature to obtain a homogeneous solution. Subsequently, the solution was subjected to ultrasonication using an ultrasonic processor (Bandelin Sonopuls UW 200, BANDELIN electronic GmbH & Co. KG, Berlin, Germany) at 25 °C for 1 h to improve polymer dispersion and eliminate air bubbles. Following this step, PLGA/HA nanofiber membranes (2 × 2 cm dimensions) were produced using the electrospinning method shown in Figure 22, which is a widely used technique for fabricating nanofibrous structures with controllable morphology and functional properties [74].

**Figure 23:**
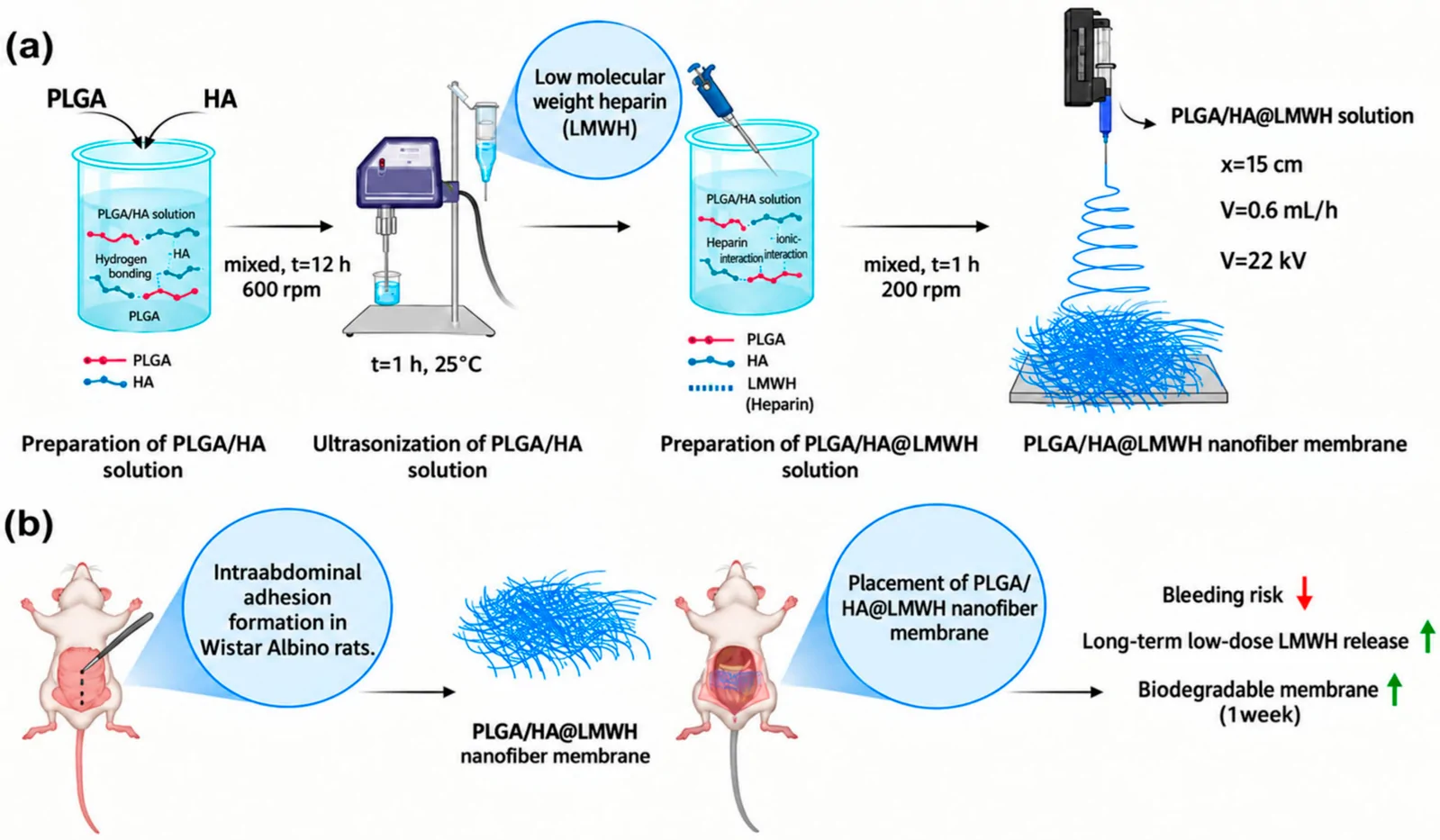
(**a**) Preparation steps of the PLGA/HA@LMWH nanofiber membrane using the electrospinning method. (**b**) Application of the prepared PLGA/HA@LMWH nanofiber membrane in a rat model to prevent intra-abdominal adhesions. The red downward arrow (↓) indicates a reduction in bleeding risk, whereas the green upward arrows (↑) indicate enhanced long-term low-dose LMWH release and membrane biodegradability.

To impart anti-adhesion and anticoagulant properties, 1000 units of LMWH (250 units of LMWH per cm2 of the nanofiber membrane) were added to the PLGA/HA solution before the electrospinning process and homogenized, resulting in PLGA/HA@LMWH nanofiber membranes. It has been stated in the literature that LMWH regulates the biological response by reducing thrombosis in nanofiber systems [75]. The amount of LMWH was determined according to the appropriate concentration that would optimize process stability, fiber morphology and biological activity. The PLGA/HA@LMWH solution was loaded into a 2.5 mL syringe with a 0.80 × 38 mm needle and electrospun at a flow rate of 0.6 mL/h, 22 kV voltage and a distance of 15 cm. All electrospinning experiments were carried out under controlled ambient conditions at a temperature of 25 ± 1 °C and a relative humidity of 50–55%. The fabrication process of PLGA/HA@LMWH nanofiber membranes is shown in Figure 23.

#### 4.2.3. Characterization Techniques

The structural and morphological properties of the nanofibers were characterized for comparison. FT-IR spectra were recorded using a Perkin-Elmer spectrophotometer in the wavelength range of 400–4000 cm^−1^. The surface morphology of the samples was examined using SEM. EDX analysis of the structures was performed using an EDX detector (model 125 eV; Bruker AXS, Berlin, Germany). XRD patterns were obtained using an X-ray diffractometer (Rigaku RadB-Dmax II model, The Woodlands, TX, USA) with Cu Kα (λ = 1.54 A°) radiation at 40 kV and 40 mA. The range of diffraction angle 2θ was 5–80° using a scanning speed of 6°/min. Furthermore, the water contact angles were measured using an optical contact angle meter (SEO Phoenix 300, Suwon, Republic of Korea). The absorbance measurements were detected with a BioTek Eon microplate reader (BioTek Instruments, Winooski, VT, USA).

#### 4.2.4. In Vitro Heparin Release

For quantitative determination of the heparin release of the electrospun PLGA/HA@LMWH nanofiber membrane, toluidine blue (TB) dye assay was used [76]. Briefly, a 0.04% (*w*/*v*) solution of TB was prepared in 0.01 M HCl/0.2% NaCl. A 2 × 2 cm^2^ PLGA/HA and PLGA/HA@LMWH nanofiber membranes were placed in a tube containing 5 mL of PBS (50 mM, pH 7.4), and the tubes were incubated in an orbital shaker (200 rpm) at 37 °C to simulate physiological conditions. At predetermined time intervals, 1.0 mL of release media was withdrawn and replaced with fresh buffer. In total, 150 µL of TB solution was added to the release media, and the final mixture was briefly vortexed. It was then incubated at room temperature in the dark for 15–20 min to allow for the complete precipitation of the TB with LMWH precipitate. Afterward, the TB formed with LMWH precipitate was centrifuged at 5000 rpm for 10 min; it was rinsed with 0.01 M HCl/0.2% NaCl and dissolved in a mixture solution of 1 mL with ethanol/0.1 M NaOH (*v*/*v* = 4/1). Finally, the absorbance of solutions was monitored using a BioTek Eon microplate reader (BioTek Instruments, Winooski, VT, USA) at 530 nm. A calibration curve was constructed using LMWH solutions of known concentrations under identical experimental conditions. The amount of LMWH released from the membranes at each predetermined time point was calculated using the corresponding calibration equation. Subsequently, the total amount of released LMWH was plotted against time to evaluate the release profile. All measurements were performed in triplicate (*n* = 3).

#### 4.2.5. Cell Viability

In total, 0.1 g was taken from each electrospun fiber and placed in 1.0 mL of high-glucose DMEM containing 10% FBS and 1% Pen–Strep. Samples were incubated at 37 °C in 5% CO_2_ for 3 days; the fibers were removed and the conditioned supernatant was collected. L-929 cells were seeded into 96-well plates (10,000 cells/well) and allowed to adhere; the medium was changed and 100 µL of conditioned DMEM was added to each well. Cells were incubated for 24 h; their morphology was examined under an inverted microscope prior to MTT. The medium was removed and DMEM containing 10% MTT was added. An MTT solution prepared in PBS at a concentration of 5 mg/mL and adjusted to pH 7.4 was used, and the plates were incubated for 4 h. At the end of the incubation period, the medium containing MTT was carefully aspirated. To dissolve the formed formazan crystals, 100 µL of DMSO was added to each well; the absorbance values of the samples were measured spectrophotometrically at a wavelength of 540 nm.

### 4.3. Experimental Animals and Ethical Approval

The study was initiated after approval from the İnönü University Faculty of Medicine Local Ethics Committee for Experimental Animals (HAYDEK, 2023/7-1; HAYBİS No: 18167). Experimental animals were housed at a temperature of 22–24 °C, 50–60% humidity, and a 12-h light/dark cycle, with ad libitum access to food and water. A total of 58 Wistar Albino female rats, aged 8–12 weeks and weighing 150–250 g, were used in the study (18 rats for the preliminary study; 40 rats for the main study).

### 4.4. Experimental Groups (Main Study)

#### Power Analysis Report and Groups

For alpha = 0.05, power = 0.8 and effect size = 0.6, at least 9 samples per group (total 36) are required to detect significant differences using one-way ANOVA in independent samples. Calculations were performed using WSSPAS (Web-Based Sample Size & Power Analysis Software, Version 2.0) [77]. The number of animals increased from 36 to 40 to account for potential losses. The main study used 40 Wistar Albino female rats, divided into four groups of 10 animals each.

− PLGA/HA Group: PLGA/HA membrane applied.
− PLGA/HA@LMWH Group: PLGA/HA@LMWH membrane applied.
− Saline Solution Group (SS): 0.9% NaCl solution applied.
− Laparotomy Group (LO): Laparotomy performed only.

### 4.5. Creating an Intraabdominal Adhesion Model

#### 4.5.1. Anesthesia and Postoperative Analgesia

All surgical procedures were performed under general anesthesia with 75 mg/kg ketamine and 8 mg/kg xylazine [78]. For postoperative analgesia, 0.01–0.1 mg/kg buprenorphine was administered subcutaneously every 12 h for 3 days [79].

#### 4.5.2. Surgical Technique

All rats underwent midline laparotomy (3 cm) after abdominal wall shaving and disinfection under ketamine–xylazine anesthesia. In the first three groups, abrasion was performed on the antimesenteric surface of the cecum in a 1.5 × 1.5 cm area using a sterile toothbrush until petechial bleeding occurred. In the PLGA/HA group, a PLGA/HA nanofiber membrane was applied to the abrasion area; in the PLGA/HA@LMWH group, a PLGA/HA@LMWH nanofiber membrane (2 × 2 cm dimensions) was applied; and in the SS group, 5 mL of 0.9% NaCl was applied. Laparotomy was performed only in the LO group. The anterior abdominal wall was closed separately with 3/0 prolene and the skin with 3/0 silk. The animals were sacrificed on day 21 with a high dose of anesthesia (four times the dose of ketamine and xylazine anesthetics).

### 4.6. Evaluation Parameters

#### 4.6.1. Macroscopic Adhesion Scoring

The abdominal cavity was opened in a U-shape, and adhesions were macroscopically evaluated by an impartial surgeon using the Nair scoring system [44] (Table 1).

#### 4.6.2. Histopathological Evaluation

Sections obtained from paraffin-embedded tissues were stained using Masson’s trichrome staining method and histopathological examinations were performed. Adhesions and inflammation, fibrosis, and vascular proliferation in surrounding tissues were evaluated. Cecum samples were fixed with 10% buffered formalin, cut from paraffin blocks, and stained with H&E, Masson’s trichrome, and immunohistochemistry. Evaluation was performed on a scale of 0–3 over 5 randomly selected fields under light microscopy according to the scoring system of Giusto et al. [80] (Table 2). Collagen density in the adhesion area was semi-quantitatively evaluated using Masson trichrome (Bio-Optica, Milan, Italy; Cat No: 04-010802) staining. Briefly, it was scored as follows: 0: no collagen; 1: less than 5% collagen in the adhesion area; 2: between 6–20% collagen; 3: more than 21% collagen.

#### 4.6.3. Immunohistochemical Evaluation

Sections were stained using the Avidin–Biotin Complex (ABC) method with TGF-beta1 antibodies (Invitrogen) [81]; the manufacturer’s (Thermo Fischer Scientific, Waltham, MA, USA) protocol was followed in the procedures. Tissues on positively charged slides were deparaffinized after being kept at 65 °C for 1 h, passed through a graduated alcohol series and brought to distilled water. To recover antigens, sections were microwaved (600 W, 15 min) in citrate buffer, cooled to room temperature, and washed with PBS. To eliminate endogenous peroxidase activity, tissues were treated with 3% H_2_O_2_ prepared in methanol for 10 min and, after PBS washing, stained according to a commercial kit protocol. After non-specific binding was inhibited with protein block (Ultra V Block) serum (10 min), sections were incubated overnight at +4 °C with TGF-beta1 primary antibody (1/200). Non-immune serum was used as a negative control. Subsequently, biotin-containing secondary antibody (30 min) and HRP (Horseradish Peroxidase)–streptavidin (20 min) were applied to the sections. Following PBS washing (15 min), sections were incubated with 3-amino-9-ethylcarbosol (AEC) (Thermo Fischer Scientific, Waltham, MA, USA) in a controlled manner and washed with distilled water. For counterstaining, sections were held in Gill’s hematoxylin solution for 45–60 s. Sections sealed with water-based adhesive (Lerner Laboratories, Philadelphia, PA, USA) were examined under a light microscope. For immunohistochemical scoring, positive cells in 5 randomly selected fields at 40× magnification were graded as 0 (none), 1 (<10), 2 (11–20), and 3 (>21).

#### 4.6.4. Biochemical Evaluation

Protein levels associated with inflammatory and apoptotic pathways (Bcl-2, cleaved caspase-3, NFκB, p-NFκB p65) in tissues obtained from abrasion sites in rats were evaluated using the Western blot method. Additionally, levels of hydroxyproline and some oxidant–antioxidant parameters in the tissues were measured.

### 4.7. Statistical Analyses

The Shapiro–Wilk normality test was applied to evaluate the distribution characteristics of the research data set. Since the data did not show a normal distribution, the non-parametric Kruskal–Wallis test was used to examine the data set, and Dunn’s post hoc test was used for intergroup comparisons. Statistical results are presented as median (minimum–maximum) values. *p* < 0.05 was considered statistically significant.

## 5. Conclusions

This study demonstrated that composite membranes obtained by adding low-molecular-weight heparin to electrospinning-produced PLGA/HA nanofiber membranes were effective in preventing intraabdominal adhesions in a rat model. This approach, combining a physical barrier and pharmacological modulation, is promising in preventing postoperative adhesions. However, more comprehensive preclinical and clinical studies are needed for clinical application.

## Figures and Tables

**Table 1:** Nair Adhesion Scoring System.

Score	Definition
0	No adhesions
1	Single band adhesion (between organs or between an organ and the abdominal wall)
2	Two band adhesions
3	More than two bands or mass adhesions between bowel loops, but no spread to the abdominal wall
4	Widespread, dense adhesions; bowel loops adhering to the abdominal wall

**Table 2:** Histopathological scoring system of Giusto et al. [80].

Grade	Inflammatory Cell Infiltration	Fibrosis	Vascularization
0	None	None	None
1	Mild	Mild	One or two vessels
2	Moderate	Moderate	Three to nine vessels
3	Severe	Intense	Ten or more vessels

## Data Availability

All data generated or analyzed during this study are included in this published article.
